# A positive feedback loop involving the Spa2 SHD domain contributes to focal polarization

**DOI:** 10.1371/journal.pone.0263347

**Published:** 2022-02-08

**Authors:** Michael J. Lawson, Brian Drawert, Linda Petzold, Tau-Mu Yi

**Affiliations:** 1 Division of Biology and Biological Engineering, California Institute of Technology, Pasadena, CA, United States of America; 2 Department of Computer Science, University of North Carolina Asheville, Asheville, NC, United States of America; 3 Department of Computer Science, University of California, Santa Barbara, Santa Barbara, CA, United States of America; 4 Molecular, Cellular, and Developmental Biology, 3131 Biological Sciences II, University of California, Santa Barbara, Santa Barbara, CA, United States of America; Institute of Biology Valrose, FRANCE

## Abstract

Focal polarization is necessary for finely arranged cell-cell interactions. The yeast mating projection, with its punctate polarisome, is a good model system for this process. We explored the critical role of the polarisome scaffold protein Spa2 during yeast mating with a hypothesis motivated by mathematical modeling and tested by *in vivo* experiments. Our simulations predicted that two positive feedback loops generate focal polarization, including a novel feedback pathway involving the N-terminal domain of Spa2. We characterized the latter using loss-of-function and gain-of-function mutants. The N-terminal region contains a Spa2 Homology Domain (SHD) which is conserved from yeast to humans, and when mutated largely reproduced the *spa2Δ* phenotype. Our work together with published data show that the SHD domain recruits Msb3/4 that stimulates Sec4-mediated transport of Bud6 to the polarisome. There, Bud6 activates Bni1-catalyzed actin cable formation, recruiting more Spa2 and completing the positive feedback loop. We demonstrate that disrupting this loop at any point results in morphological defects. Gain-of-function perturbations partially restored focal polarization in a *spa2* loss-of-function mutant without restoring localization of upstream components, thus supporting the pathway order. Thus, we have collected data consistent with a novel positive feedback loop that contributes to focal polarization during pheromone-induced polarization in yeast.

## Introduction

Cell-cell interactions often involve thin projections. Such projections arise from focal polarization in which growth is confined to a narrow region such as a polarized tip [1, 2]. The docking of slender projections results in precise cell-cell contacts (e.g. neural synapses [3]). Other examples include cell-cell and cell-matrix interactions involving focal adhesions during cell migration [4] or formation of the immunological synapse [5]. The precise mechanisms for achieving focal polarization remain to be elucidated.

**Fig 1:**
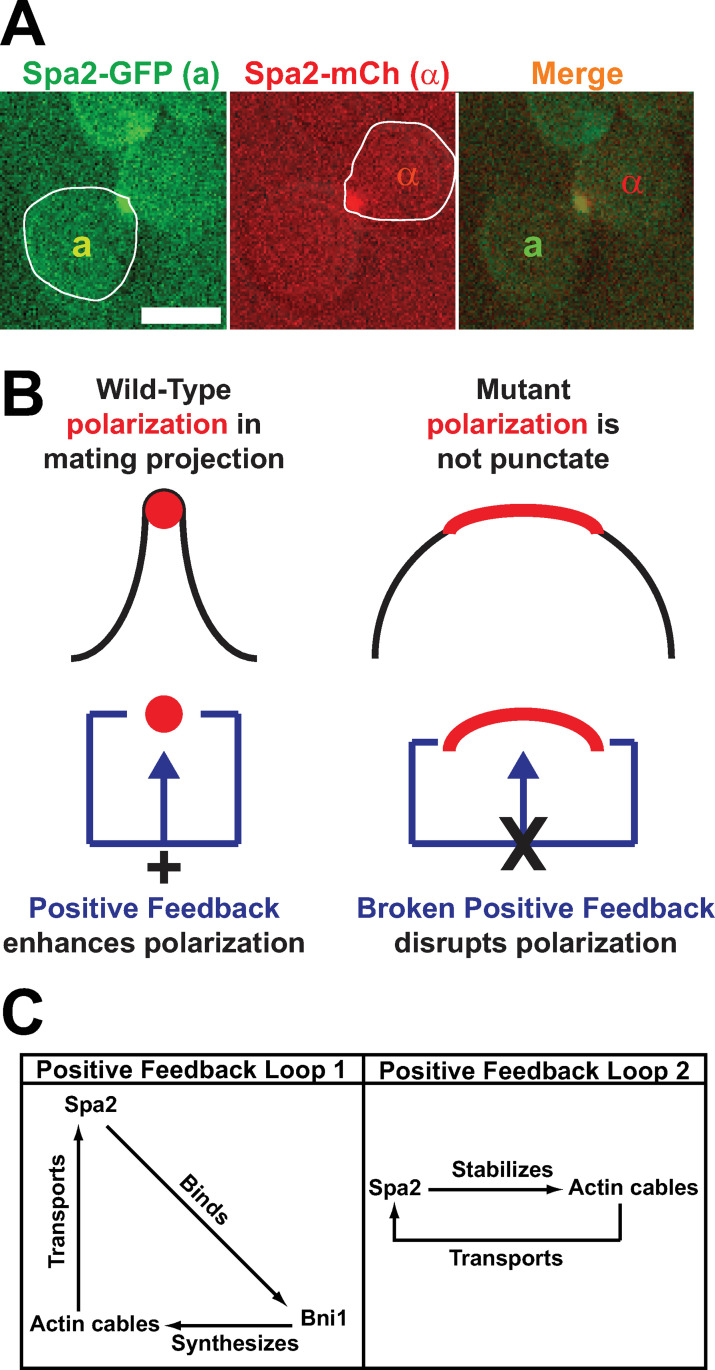
Focal polarization of polarisomes and positive feedback. **(A)** Alignment of focal polarisomes during yeast mating. Left: polarisome of **a**-cell (Spa2-GFP, left cell). Middle: polarisome of α-cell (Spa2-mCherry, right cell). Right: overlay shows colocalization. Scale bar = 5 μm. **(B)** In model, positive feedback enhances polarization resulting in wild-type punctate polarisome (left). Disruption of positive feedback results in broader polarization of polarisome proteins and wider projection morphology (right). **(C)** Schematic of two proposed positive feedback loops. In Loop 1, Spa2 recruits Bni1 to the membrane where it synthesizes actin cables along which Spa2 is transported. In Loop 2, Spa2 stabilizes actin cables which carry more Spa2 to the membrane.

Mating in the budding yeast *S*. *cerevisiae* has been a successful model system for cell-cell communication during synapse formation [6, 7]. Yeast cells have two haploid mating types **a** and α, which secrete pheromones (**a**-factor and α-factor) (Fig 1A). These secreted peptides form a spatial gradient that is sensed by the mating partner via G-protein coupled receptors [7, 8]. Receptor activation elicits a cellular response that involves cell polarization and formation of mating projections up the pheromone gradient, which meet and fuse [9].

The polarisome serves as the focal point of mating projection growth [10, 11]. It is localized at the projection tip [9, 12] and ensures that polarized transport and secretion along actin cables is targeted to a narrow region of the cell membrane. As defined previously [10], the polarisome consists of three core proteins that co-precipitate when pulled down by antibodies: Spa2, Bni1, and Bud6. Bni1 is a formin that initiates the polymerization of actin cables, which direct vesicles to the front of the cell [13]. Bud6 stimulates the catalytic activity of Bni1 [14]. Spa2 is a scaffold protein that binds together the components of the polarisome [15, 16]. In the absence of Spa2, the polarisome loses its focal appearance during mating, resulting in a wide instead of narrow projection. Mutants that exhibit abnormal polarisome dynamics also exhibit decreased mating [15, 17, 18]. One hypothesis is that proper mating requires the alignment of punctate polarisomes (Fig 1A). Rose and colleagues have demonstrated that during mating, cell wall degradation has to be correctly timed and localized [19], and polarisome components are important for delivering the cell wall hydrolases to a specific spot allowing piercing while maintaining cell integrity elsewhere. A fourth polarisome protein is the Spa2 homolog Pea2 which binds and is stabilized by Spa2 [20, 21].

Structure-function studies have been performed on Spa2 [10, 15]. Deletions of the C-terminal end revealed a region that interacts with Bni1 [22]. A 254 amino acid C-terminal region of Spa2 (1213–1466) interacted with Bni1 in both two-hybrid and pull-down experiments. However, mutants possessing Spa2 C-terminal truncations to residue 1074 or even further to 655 exhibited wild-type mating and Spa2 localization suggesting that this C-terminal interaction with Bni1 is not critical for mating function [15]. The N-terminal domain contains an evolutionarily-conserved Spa2 Homology Domain (SHD) which consists of two copies of a 31 residue Spa2 direct repeat (SDR). Deletion of this domain results in defective mating [15]. The conserved domain plays an essential role in other eukaryotes as well. For example in the mammalian Arf GAP GIT1, a conserved SHD domain interacts directly with Pix, a Rac GEF, and with Piccolo, a core member of the neuronal cytoskeletal matrix assembled at the active zones of neural synapses [23].

Previous two-hybrid results revealed that the SHD domain of Spa2 interacted with Msb3 and Msb4 [24], which are GTPase-activating proteins (GAPs) for Rab G-proteins in yeast including Vps21 in endosomes and Sec4 on secretory vesicles. This binding is responsible for Msb3/4 localization to sites of polarized growth. Deleting both genes resulted in less polarized actin organization during budding [25]. They are members of the larger Gyp family of Rab GAPs, several of which are known to act on Sec4 *in vitro* including Gyp2, Msb3, and Gyp8 [26].

Sec4 is a small G-protein of the Rab class that mediates the transport of vesicles from the Golgi to the plasma membrane [27]. Loss-of-function mutants of Sec4 such as the temperature-sensitive *sec4-8* mutation are defective in secretion [28]. Walworth and colleagues [29] characterized the *sec4*^*Q79L*^ mutation that exhibits impaired GTP hydrolysis. In addition, *sec4* mutants affect actin cable formation and Bud6 localization [30]. Sec3 is also involved in the secretory pathway as a component of the exocyst mediating the docking of secretory vesicles to the plasma membrane. It co-localizes with Sec4 [31] and the polarisome [16] at sites of polarized exocytosis.

Bud6 and Bni1 represent the other two main components of the polarisome. Bud6 is a nucleation promoting factor for the formin Bni1, stimulating actin cable formation [32]. Bud6 interacts with the C-terminus of Bni1, neutralizing its auto-inhibitory properties [33]. While a *bud6Δ* mutant still forms a bud, it shows a marked decrease in actin cables. It is important to note that most of the results in the literature on these polarization proteins focused on budding and not mating [34]. Taken together, the above research outlines a pathway from Spa2 to Msb3/4 to Sec4 to Bud6 during budding.

While this paper was under review, Dünkler et al. [35] demonstrated that an interaction between the polarisome component Pea2 and the type V Myosin Myo2, which is involved in actin-based polarized transport of secretory vesicles, A mutation that disrupted this interaction impaired polarization of the polarisome during budding, and produced round instead of elongated buds. We highlight the connections between this new research and our results in the Discussion.

In previous work we introduced the concept of spatial amplification, which refers to tighter polarization than the spatial input cue and is exemplified by the punctate cohesion of the polarisome during mating [16]. We constructed a stochastic model of the polarisome that reproduced the *spa2Δ* phenotype of decreased polarization and the appearance of dispersed polarisome mini-clusters. In our model, two positive feedback loops contributed to the narrow polarization (~20°); one involved the previously described [22] direct binding of Spa2 to Bni1 to recruit more Bni1 to the membrane. A second novel positive feedback loop involved Spa2 stabilizing actin cables, thereby facilitating transport of more Spa2 to that location.

In this work, we have used mathematical modeling as a starting point to better understand Spa2-dependent focal polarization during yeast mating. We have found experimental evidence that the Spa2 N-terminal SHD domain recruited Msb3 and Msb4, which then act through Sec4 for proper polarization of Bud6, stimulating actin cable formation by Bni1. Gain-of-function modifications demonstrated that forced polarisome localization of the SHD domain, Msb3, and the Bud6 catalytic domain partially offset *spa2* loss-of-function. These results combined with previous results in the literature are consistent with a novel positive feedback pathway that contributes to focal polarization during yeast mating.

## Results

### Simulations predict Spa2 promotes focal polarization via two positive feedback loops

The mating projection of a *spa2* mutant is much wider than that of a wild-type cell [12]. The goal of this work is to elucidate how Spa2 contributes to focal polarization and a narrow projection during the mating response of wild-type cells (Fig 1B). Previously, we investigated Spa2 during pheromone-induced polarization, and in particular, the spatial dynamics of the polarisome, a structure that directs transport of proteins and lipids to the projection tip [16]. Spa2 is a polarisome scaffold protein, and in *spa2Δ* cells, the polarisome is broader and more dynamic. Note that the morphology defect is not caused by an alteration in pheromone sensitivity; the *spa2Δ* mutant cells possess the same sensitivity to α-factor as wild-type cells in a halo assay (S1 Table).

We proposed that two positive feedback loops are necessary to form a focal, punctate polarisome producing the narrow wild-type projection: 1) Loop 1 in which Spa2 recruits the formin Bni1 to the membrane, and 2) Loop 2 in which Spa2 increases the number of actin cables along which more Spa2 is transported [36] to the polarisome (Fig 1C). We used simulations to explore the relationship between the *spa2* phenotype and these positive feedback loops.

In this work we introduced two metrics to quantify polarisome phenotypes: (1) autocorrelation measured the stability of the polarisome (see Materials and Methods), and (2) full-width at half-maximum (FWHM) described the width of the polarisome. We examined four versions of the model: Both positive feedback loops (WT); Loop 1 only (Spa2-L1); Loop 2 only (Spa2-L2); and neither loop (spa2Δ).

We observed that the autocorrelation of the WT model was higher than that of the *spa2Δ* model, reflecting the increased dynamic behavior of the latter (S1 Fig). The Spa2-L1 and Spa2-L2 simulations exhibited intermediate autocorrelation between WT and spa2Δ.

**Fig 2:**
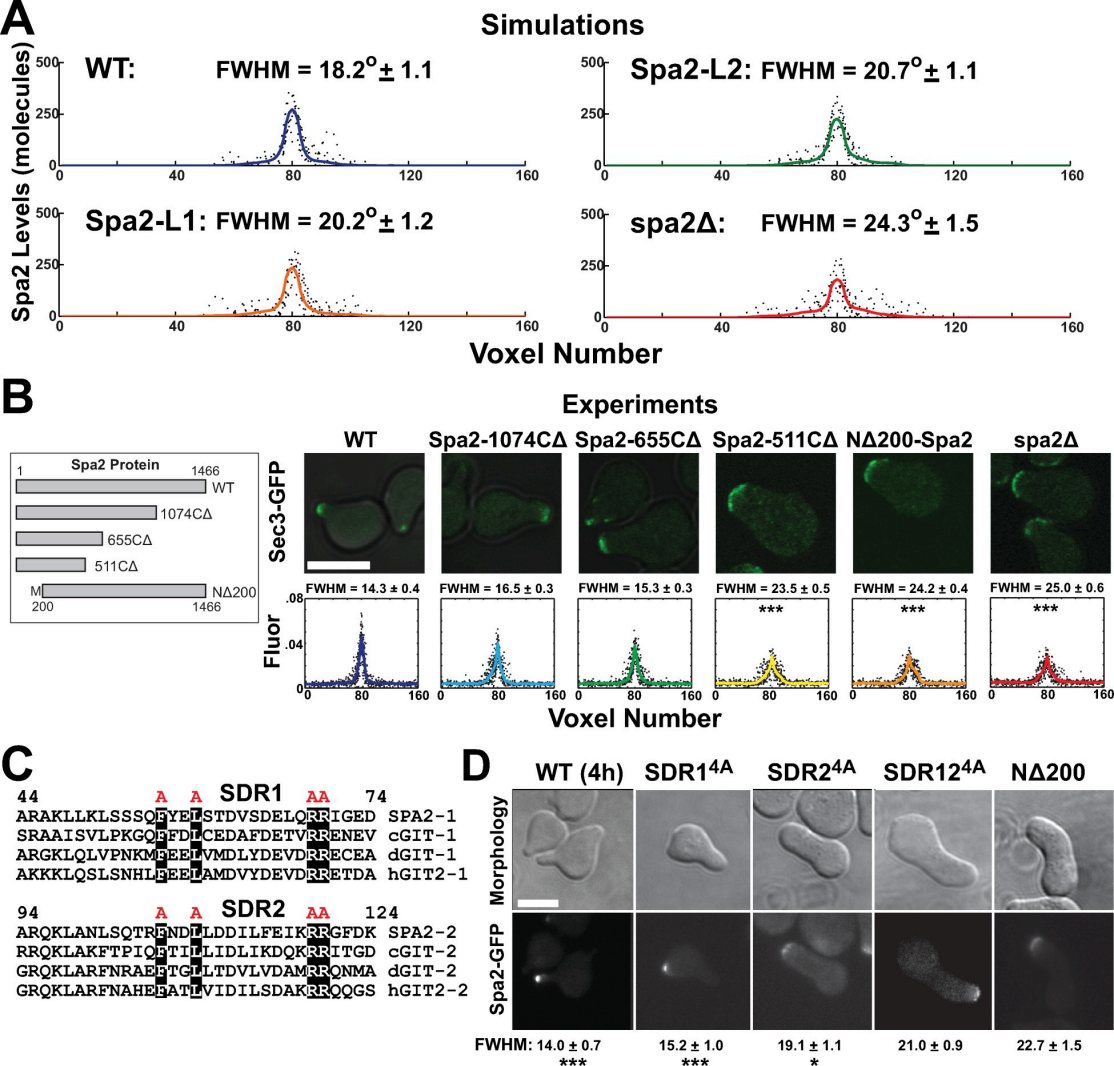
Polarisome phenotypes of *spa2* mutants. **(A**) Full width at half maximum (FWHM) measurements for simulated Spa2 partial deletions. Four types of simulations were run: WT (top row, blue), Spa2-L1 with Loop 1 only (orange), Spa2-L2 with Loop 2 only (green), and spa2Δ (bottom row, red). The solid lines indicate average Spa2 polarization from 100 simulations (used to calculate the FWHM); dotted lines show profiles from individual simulations. The cell perimeter was divided into 160 voxels, and the numbers of molecules per voxel are shown on the *y*-axis. FWHM in degrees is calculated from plot. **(B)** Polarisome morphologies and *in vivo* FWHM measurements for Spa2 partial deletions. Left: schematic diagram of Spa2 deletion constructs. Right: Sec3-GFP marks the polarisome in cells treated with pheromone for 2h. Top row shows overlaid fluorescent and bright-field images, and bottom row shows average spatial distribution of Sec3-GFP (solid line) for 3 experiments of 8 cells each (dotted black). The cell perimeter was divided into 160 voxels, and the normalized fluorescence per voxel is shown on the *y*-axis. Scale bars = 5 μm in figure. **(C)** Sequence alignment of SDR repeats. The sequences of the two Spa2 Direct Repeats (SDR1 and SDR2) in Spa2 and the GIT proteins from *C*. *elegans* (cGIT), *Drosophila* (dGIT), and humans (hGIT2) are aligned showing the conserved amino acids. The four highlighted residues were mutated to alanine. Numbering is respect to Spa2 protein sequence. **(D)** Polarisome phenotypes in SDR mutants. The four alanine substitution was made in *SDR1*^*4A*^, *SDR2*^*4A*^, and in both *SDR12*^*4A*^. The mutated Spa2 was tagged with GFP and visualized after 4h treatment with α-factor. FWHM measurements underneath images were made for Spa2-GFP in all strains (n = 20 cells), which were compared to *NΔ200-spa2* by t-test (***, p < 0.001; *, p < 0.05).

We also measured the width of the polarisome by calculating the average FWHM of 100 simulations for each model. The polarisome in the WT model was narrower and more focal than that of *spa2Δ* (18.2° versus 24.3°). Once again, the Spa2-L1 and Spa2-L2 simulations displayed an intermediate phenotype (Fig 2A). Thus, the simulations suggested that both positive feedback loops are necessary for a stable focal polarisome. Although Loop 1 was based on experimental information from the literature, Loop 2 represented a novel predicted pathway. We wished to identify and experimentally characterize Loop 2.

### Deletion and point mutations of Spa2 affect polarisome polarization

The first step was to perform structure-function analysis to identify regions in Spa2 that disrupted wild-type function resulting in a mutant (broad) mating projection. It is known that the C-terminus of Spa2 binds Bni1 [22], and this interaction forms the basis of Positive Feedback Loop 1. We wished to investigate the impact of disrupting this loop through a Spa2 C-terminal deletion. In addition, we hypothesized that the N-terminal portion of Spa2 may be involved in Loop 2. Thus, dissection of this region could pinpoint key binding motifs and facilitate the search for binding partners that would help elucidate the Loop 2 pathway.

#### N-terminal Spa2 deletion resembles *spa2Δ* phenotype

Previous work demonstrated the broad polarization of the mating projection in *spa2* mutants [10, 12, 15]. We examined the loss of focal polarization in greater detail by constructing N- and C-terminal deletions, and assessing the localization of polarization markers.

The C-terminal Spa2 deletions to positions 1074 and 655 both removed the domain that binds Bni1 [22]. The mutants exhibited a mild phenotype with slightly broader projections and wider polarisomes after 2h treatment with pheromone (Fig 2B). Deleting to position 511 resulted in the more dramatic *spa2Δ* phenotype, caused by the absence of Spa2 near the membrane (S2 Fig). Thus, abrogating Loop 1 had only a modest effect on polarization consistent with data that the C-terminal domain of Spa2 is not necessary for mating [15].

One the other hand, the N-terminal *NΔ200-spa2* mutant produced a phenotype similar to *spa2Δ* with a single broad peanut-shaped mating projection at 2h of pheromone treatment (Fig 2B). However, unlike in *spa2-511ΔC and spa2Δ*, Spa2 (NΔ200-Spa2) was present near the membrane in the *NΔ200-spa2* mutant, but did not form a punctate polarisome, instead appearing in dispersed polarisome mini-clusters characteristic of *spa2Δ* polarization. We calculated the full-width at half-maximum (FWHM) of the Sec3-GFP (which co-localizes with the polarisome) polarization and found that it was significantly wider for *NΔ200-spa2* (24.2°) and *spa2Δ* (26.0°) phenotypes compared to wild-type (14.3°) reflecting the morphological differences. Thus, *NΔ200-spa2* and *spa2-511ΔC* showed broad polarization and projections similar to *spa2Δ*; each represents the loss of the N-terminal domain either through deletion or absence of the Spa2 protein. The key point is that NΔ200-Spa2 is still at the membrane, and yet the polarisome is dispersed, indicating a strong role for the Spa2 N-terminus.

The wild-type (WT) and *spa2Δ* polarisome widths (Fig 2B) marked by Sec3-GFP were roughly similar to the values in the simulations. Interestingly, the *NΔ200-spa2* mutant had a more pronounced (i.e. less polarized) phenotype than predicted by simulations lacking Loop 2.

We also examined the polarization of Bni1 in the *spa2* mutants. The coupled interactions of Bni1 and Spa2 via the positive feedback loops play a critical role in polarisome dynamics. We quantified the width of Bni1 localization in the various *spa2* strains (S3 Fig). Consistent with the Sec3-GFP data, Bni1-GFP was more broadly distributed in the *NΔ200-spa2* and *spa2Δ* cells compared to wild-type. The *spa2-655ΔC* strain exhibited a slightly wider Bni1 polarization from wild-type indicating a milder morphological defect compared to *spa2Δ*.

#### Mutational analysis of Spa2 SDR motifs

The next step was a more detailed characterization of the Spa2 N-terminus. Our focus was on a conserved sequence found in this region. A protein segment termed the Spa2 Homology Domain (SHD) encompasses the N-terminal 150 amino acids of Spa2 (removed in *NΔ200-spa2*), and contains two Spa2 Direct Repeats (SDRs) [15]. Previous work in rat [23] identified four highly-conserved amino acids from yeast to human in this motif (Fig 2C, black highlighted). We mutated these residues to alanine in each repeat (*SDR1*^*4A*^ and *SDR2*^*4A*^) and in both repeats (*SDR12*^*4A*^) to test whether perturbing these specific residues in the SDR domains would have a phenotypic effect. These mutations have not been characterized in yeast.

The tandem repeat mutant (*SDR12*^*4A*^) produced Spa2 but exhibited a projection morphology and polarisome phenotype similar to the *NΔ200-spa2* and *spa2Δ* mutants. The single repeat *SDR1*^*4A*^ mutant revealed a partial polarization defect, whereas *SDR2*^*4A*^ possessed a phenotype similar to but less severe than *SDR12*^*4A*^ and *spa2Δ* (Fig 2D). We performed quantitative analysis and found both the projection and the polarisome in the *SDR2*^*4A*^ and *SDR12*^*4A*^ mutants to be significantly wider than in wild-type. The cells were treated with α-factor for 4h to highlight the morphological differences from wild-type.

In summary, the N-terminal deletion of Spa2 had a more pronounced effect on projection morphology and polarisome localization than the C-terminal deletions. Site-directed mutagenesis localized the key N-terminal motif to two SDR repeats within the SHD that are conserved in eukaryotes. The *SDR12*^*4A*^ mutant largely reproduced the N-terminal deletion phenotype, as well as the full *spa2Δ* deletion. A key question is what are the components downstream of Spa2 SHD that are responsible for producing the wild-type narrow mating projection/polarisome morphology.

### Actin and secretion defects in *spa2* mutants

Our modeling hinted at where to look next to explain the mutant polarization phenotypes. According to the model, Loop 2 (Fig 1C) involves Spa2 promoting actin cable formation by inhibiting actin depolymerization (or equivalently by stimulating actin polymerization) so that there are more cables to transport Spa2 to the projection tip. Thus, we examined the hypothesis that disrupting this positive feedback loop would decrease actin cable polarization.

#### Reduced polarization of actin cytoskeleton in N-terminal *spa2* mutant

**Fig 3:**
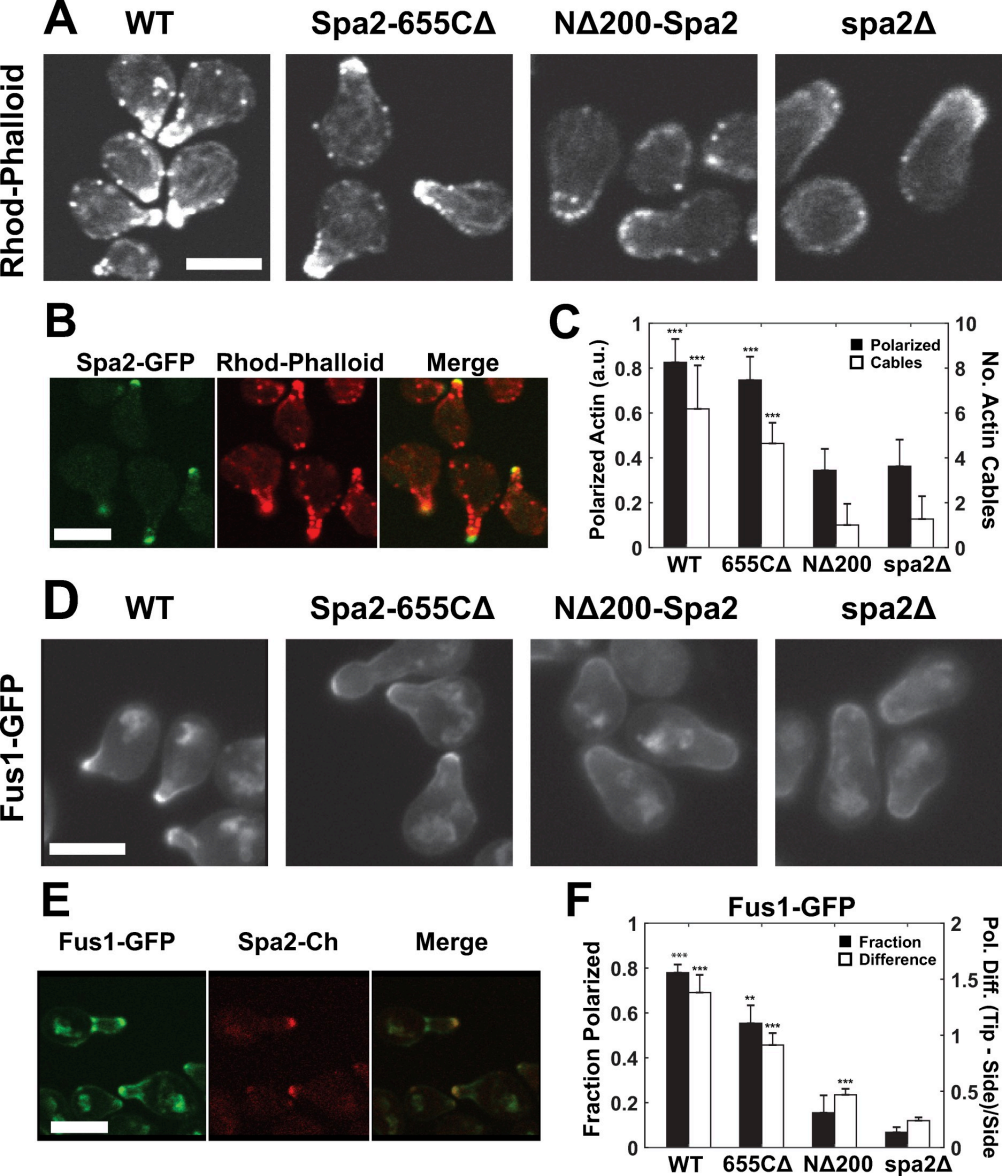
N-terminal deletion of Spa2 and *spa2Δ* exhibit actin and secretion defects. **(A)** Actin cytoskeleton in WT and *spa2* mutant strains. Cells were treated with α-factor for 2 hours, and then stained with rhodamine-phalloidin. Scale bars = 5 μm in figure. **(B)** Co-localization of actin puncta with polarisome. Cells containing Spa2-GFP were treated with α-factor for 2 hours, and then co-stained with rhodamine-phalloidin. Individual and merged images are shown. In projection at bottom, polarisome is slightly closer to the cell surface than actin puncta. **(C)** Quantification of polarized actin in WT and *spa2* mutant strains. The average intensity per pixel of rhodamine-phalloidin fluorescence in the mating projection was quantified in each strain (***, p < 0.001 by t-test; n ≥ 20 cells); mean and SD are shown (black bars). Number of actin cables in projection (***, p < 0.001 by t-test; n ≥ 20 cells); mean and SD are shown (white bars). In t-test, comparison is to *spa2Δ*. **(D)** Polarization of Fus1-GFP in WT and spa2 mutant cells. WT and mutant cells containing Fus1-GFP were treated with α-factor for 2 hours, and then imaged. **(E)** Co-localization of Fus1-GFP puncta with polarisome. Cells containing Fus1-GFP and Spa2-mCherry were treated with α-factor for 2 hours and then imaged by confocal microscopy. Individual and merged images are shown. **(F)** Quantification of Fus1-GFP polarization in WT and *spa2* mutant strains. The fraction of cells (black bars) exhibiting polarized Fus1-GFP fluorescence in the mating projection, and the relative difference (white bars) between projection tip fluorescent intensity and projection side intensity, i.e. (tip–side)/side = tip/side– 1, were calculated. Data (mean and SD) are shown from 3 trials (at least 20 cells per trial) for each strain (**, p < 0.01; ***, p < 0.001 by t-test; n ≥ 20 cells). In t-test, comparison is to *spa2Δ*.

To test for the presence of the proposed second positive feedback loop, we examined whether *spa2* mutations affected the actin cytoskeleton during the pheromone response. In Fig 3A, we visualized the actin cytoskeleton with rhodamine-phalloidin, which stains polymerized actin (cables and patches), after 2h of pheromone treatment. The *spa2Δ* mutant revealed fewer actin cables overall, and less polarized actin staining compared to wild-type (WT). These data are consistent with findings that actin patch polarization is reduced in *spa2Δ* mutants during yeast spore germination preceding budding [37].

More specifically, WT cells possessed strongly staining actin puncta that colocalized with (or were slightly interior to) the polarisome at the tip of the projection (Fig 3B); these were less pronounced in *spa2Δ*. The C-terminal *spa2-655CΔ* deletion had an actin staining pattern similar to WT, whereas the N-terminal *NΔ200-spa2* deletion closely resembled *spa2Δ* (Fig 3A). Quantification of fluorescence intensity and the number of actin cables in the projection showed that the polarized actin distributions in the WT and C-terminal deletion strains were significantly higher than in the *spa2Δ* or *NΔ200-spa2* strains (Fig 3C). Thus, pheromone-induced actin polarization is reduced in *spa2* mutants that lack the N-terminal SHD domain.

#### Secretory pathway defects in N-terminal *spa2* mutant

The close connection between the actin cytoskeleton and polarized secretion suggested the hypothesis that perturbing Spa2 may also disrupt secretion. Mutations in the secretory pathway (i.e. Sec4) possess an abnormal actin cytoskeleton [38]. In addition, *spa2Δ* cells did not localize Sec4 to the bud tip during budding [21]. To determine whether the secretory pathway was disturbed in *spa2* mutants during mating, we investigated the spatial patterning of a marker of the secretory pathway, the pheromone-induced polarization protein Fus1. The distribution of Fus1 depends on the exomer complex, which involves Sec4-mediated transport [39].

Fus1 localization during the pheromone response showed a defect in *spa2Δ* and *NΔ200-spa2* cells (Fig 3D), following a similar pattern as the actin staining (Fig 3A). In WT cells, Fus1 had a punctate appearance that coincides with the polarisome (Fig 3E). In *spa2Δ* cells, it was less abundant and not as tightly polarized in the mating projection. The C-terminal *655CΔ* mutant possessed punctate Fus1-GFP localization similar to WT, whereas Fus1-GFP was dispersed in the *NΔ200-spa2* mutant as in *spa2Δ*. Quantification of the fraction of cells with polarized Fus1-GFP and the degree of polarization at the tip confirmed the decreased Fus1 polarization in the *NΔ200-spa2* and *spa2Δ* mutants (Fig 3F).

In summary, as predicted by the model, *spa2* loss-of-function mutations reduced actin polarization, and showed defects in the localization of Fus1, whose transport to the mating projection tip depends on Sec4. Both effects were observed when the Spa2 SHD region was deleted.

### Localization of Msb3/4 to polarisome at projection tip depends on Spa2 SHD

The next step was to ascertain the species directly downstream of Spa2 SHD in the putative positive feedback pathway that affects secretion and actin polarization. Possible candidates include proteins that bind to this N-terminal region of Spa2. Tcheperegine et al. showed by two-hybrid assays that Spa2 SHD interacts with Msb3 and Msb4, and this interaction was required for clustering the two proteins at the bud tip [24]. Both belong to the Gyp family that acts as GTPase activating proteins (GAPs) for Rabs (e.g. Sec4) and Arfs in yeast [26]. Importantly, Gao et al. showed that the GAP activity of Msb3 and Msb4 was required for efficient actin organization and exocytosis during budding [40], which could help explain the actin and Fus1 localization phenotypes described in the previous section.

**Fig 4:**
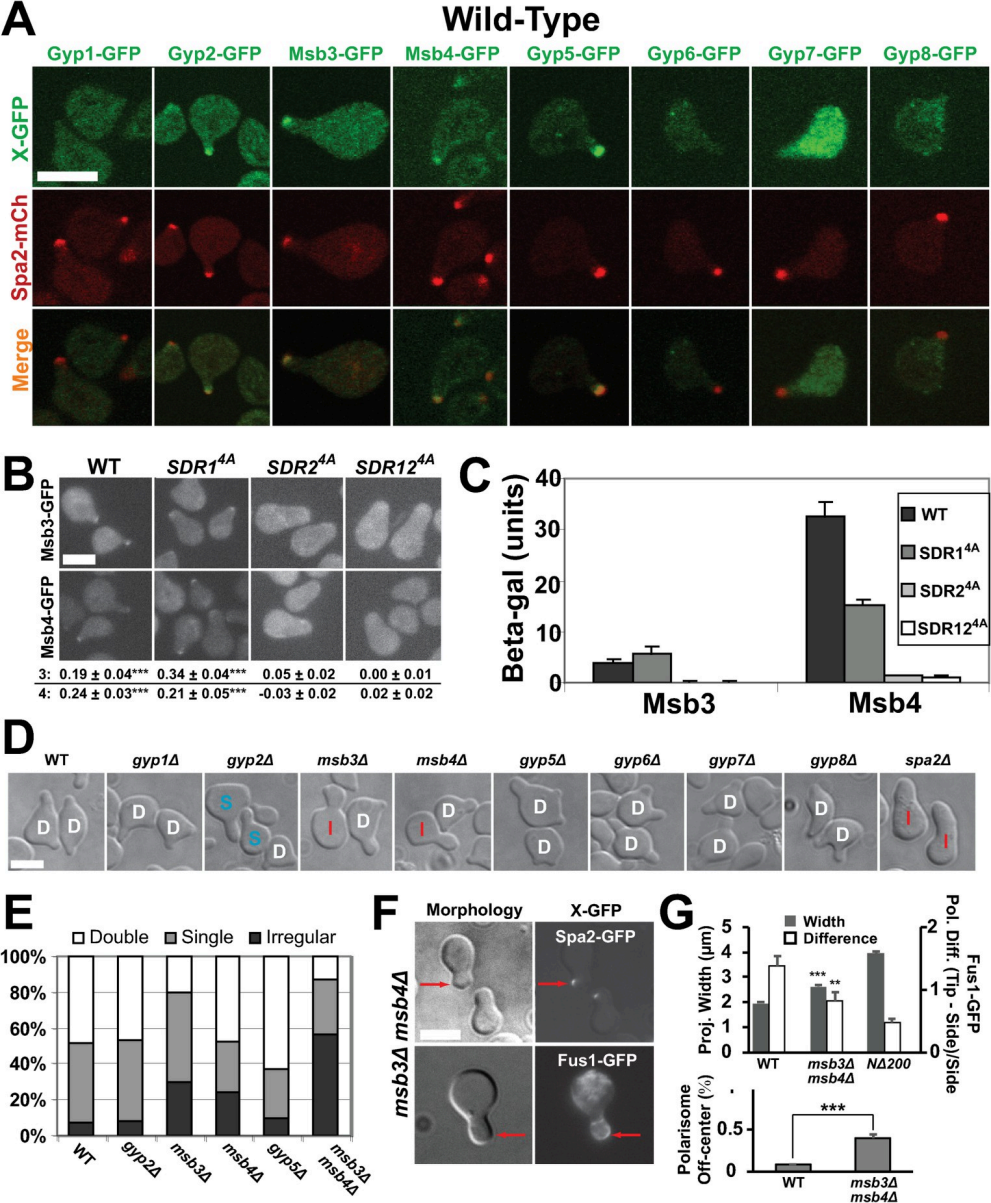
Localization of Msb/Gyp proteins and characterization of deletion mutants. **(A)** Co-localization of Msb/Gyp proteins with Spa2 in WT. Msb/Gyp proteins were tagged with GFP; Spa2 was tagged with mCherry. Cells were treated for 2h with α-factor. Scale bars = 5 μm in figure. **(B)** Localization of Msb3/4 in *SDR* mutant background. Msb3 and Msb4 were tagged with GFP in WT, *SDR1*^*4A*^, *SDR2*^*4A*^, and *SDR12*^*4A*^ strains. Imaging was performed after 2h α-factor treatment. Quantification below panel is polarization fluorescence ratio between tip versus cytoplasm indicating extent of polarization (***, p < 0.001 by t-test; n = 20 cells). Mean and SE are shown. In t-test, comparison is to *SDR12*^*4A*^. **(C)** Two-hybrid analysis of WT and mutant SHD domains interacting with Msb3 and Msb4. The N-terminal regions of Spa2 (1–200), Msb3 (1–220), and Msb4 (1–144) were used. Beta-galactosidase activity was measured in Miller units. Mean and standard deviation from 3 experiments are shown. **(D)** Morphological phenotypes of single deletion mutants. Cells were treated with α-factor for 4h and then imaged (DIC). Morphologies were classified as double (D), single (S), and single irregular (I) projections. **(E)** Quantification of projection morphologies for WT, *gyp2Δ*, *msb3Δ*, *msb4Δ*, *gyp5Δ*, and *msb3Δ msb4Δ* deletion mutants treated for 4h. Projection morphology was manually characterized as single, double, and irregular projections for at least 100 cells. **(F)** Morphology and marker polarity in *msb3Δ msb4Δ* double mutant. The *msb3Δ msb4Δ* strain containing Spa2-GFP (4h) or Fus1-GFP (4h) was treated with pheromone. Arrows show Spa2-GFP and Fus1-GFP in off-center positions. **(G)** Quantification of polarization defects in *msb3Δ msb4Δ* double mutant (4h). Projection width (gray) and Fus1-GFP polarization relative difference between projection tip fluorescence intensity versus side intensity (white) are shown (top). A significant difference compared to wild-type was observed for both (***, p < 0.001; **, p < 0.01 by t-test; n = 20 cells). The polarisome was classified as off-center if greater than 0.5 μm from projection tip (bottom). Chi-squared test (df = 1) showed significantly greater fraction of mutant cells with off-center polarisome compared to WT (***, p < 0.001; n = 20 cells). Mean and SE are shown.

We examined the localization of 8 members of the Gyp family (Gyp1, Gyp2, Msb3, Msb4, Gyp5, Gyp6, Gyp7, Gyp8) during the pheromone response by tagging with GFP. As expected, both Msb3 and Msb4 localized to the mating projection tip along with Gyp2 and Gyp5; the others were cytoplasmic (Fig 4A, top row). After dual labeling, we observed that Gyp2, Msb3, Msb4, and Gyp5 colocalized with Spa2 in the polarisome (Fig 4A, bottom row).

Msb3 and Msb4 did not polarize in *spa2Δ* (S4 Fig), nor in either the *SDR2*^*4A*^ or *SDR12*^*4A*^ mutants during pheromone treatment; by contrast they were polarized in *SDR1*^*4A*^ (Fig 4B). Gyp2 and Gyp5 did not polarize in *spa2Δ*, showed polarization in *SDR1*^*4A*^, and Gyp5 showed partial polarization in *SDR2*^*4A*^ (S4 Fig). Quantification of polarization intensity revealed significant polarization in WT and *SDR1*^*4A*^ but not in *SDR2*^*4A*^ or *SDR12*^*4A*^. Thus, the Spa2 SDRs, and *SDR2* in particular, were necessary for the proper localization of Msb3/4 to the mating projection tip.

Finally, we performed two-hybrid analysis that showed wild-type SHD and *SDR1*^*4A*^ were able to bind the N-terminal Spa2 interaction domains of Msb3 and Msb4 [24], whereas SDR2^4A^ and SDR12^4A^ could not (Fig 4C), consistent with the localization data. Taken together, these results demonstrate that Spa2 SHD, and in particular *SDR2*, is necessary for localization of Msb3/4 during mating projection growth, which complements previous data [24] showing that Spa2 SHD is necessary for localization of Msb3/4 during budding.

### Mating projection morphology phenotypes of *msb3/4* and *sec4* mutations

From a genetic functional analysis standpoint, if Msb3/4 lie downstream of Spa2 SHD in a pathway for shaping the mating projection, then one would expect *msb3/4* loss-of-function mutants to display an abnormal projection phenotype. We tested this hypothesis by constructing deletions of the Msb/Gyp gene family, and then treating the mutant strains with α-factor. Previous work on this family of proteins focused on budding [24, 40]; here we have examined mating.

#### Abnormal polarization in some single and double mutants of Msb/Gyp genes

We constructed single deletions of the 8 members of the Msb/Gyp family, and examined the shape, size, and number of projections after 4h. In this work, we investigated projection morphologies at both 2h and 4h, representing an earlier and a later stage of mating projection formation. The prolonged treatment for 4h can give rise to a more pronounced mutant phenotype compared to wild-type (which makes a second projection) making morphological assessment easier. Note that typical mating assays measure mating over a 4-6h time period [41], and that although most mating occurs within the first 2h, mating can still happen at later time points as cells produce a second projection after a thwarted first projection mating.

After 4h α-factor treatment, *spa2Δ* cells continued to extend a large first projection resulting in a clear morphological difference from the double projection phenotype of WT. Among the *msb/gyp* mutants, the *msb3Δ* and *msb4Δ* deletions exhibited the most significant abnormalities, with more single or irregular projections at 4h (Fig 4D and 4E). The most unusual morphology was the “hammerhead” projection (classified as irregular “I” in Fig 4D), which displayed a broad (like *spa2Δ*) rather than narrow tip, and relatively narrow base (like WT), representing an intermediate phenotype. In addition, the *msb3Δ msb4Δ* double mutant possessed a more pronounced phenotype than any of the single deletions with a greater percentage of irregular projections (Fig 4E & 4F).

#### Polarization markers and morphological quantification in *msb3Δ msb4Δ*

To further characterize the double mutant *msb3Δ msb4Δ*, we examined the projection morphology and two polarization markers at 4h of α-factor treatment. First, the mating projection width in the double mutant was wider compared to wild-type although not as wide as *NΔ200-spa2* cells (Fig 4G). Second, Spa2-GFP showed that the polarisome was often off-center, resulting in lateral rather than axial growth, producing the hammerhead shape (Fig 4F, top row). Quantification revealed that 40% of *msb3Δ msb4Δ* cells possessed a polarisome classified as off-center (greater than 0.5 μm from center) compared to 9% for wild-type (Fig 4G, bottom). Finally, Fus1 exhibited significantly reduced polarization similar to the phenotype in *spa2Δ* or *NΔ200-spa2* mutants, and in contrast to the wild-type punctate appearance (Fig 4G, top). The decreased Fus1 polarization in the *msb3Δ msb4Δ* mutant even in the presence of Spa2 is consistent with the role of Msb3/4 on polarized transport of Fus1 downstream of Spa2. Thus, *msb3Δ msb4Δ* cells possessed intermediate polarization and morphological phenotypes between wild-type and *spa2Δ*, with a wider projection front and less focal distribution of polarisome proteins at the projection tip compared to wild-type, but less broad than *spa2Δ* cells.

#### Polarization defects in *sec4* mutant

**Fig 5:**
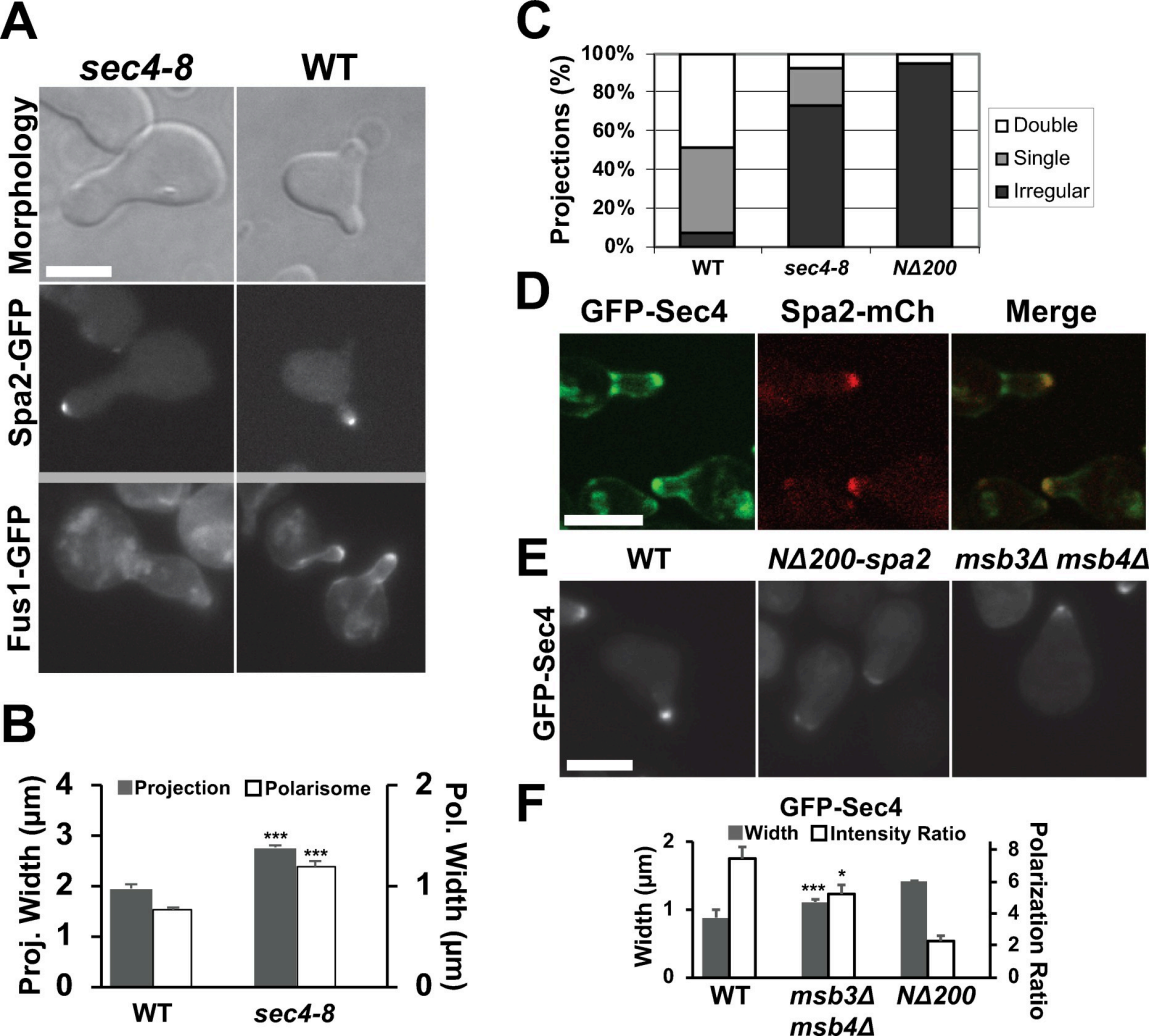
Characterization of *sec4* mutant and GFP-Sec4 localization. **(A)** Morphology (top), polarization of Spa2-GFP (middle), and Fus1-GFP (bottom) in *sec4-8* and WT cells treated for 4h with α-factor. Scale bars = 5 μm in figure. **(B)** Quantification of projection (gray) and polarisome (white) widths for *sec4* mutant and wild-type strains. Cells were treated for 4h with α-factor. Both widths for *sec4-8* were significantly wider than wild-type (***, p < 0.001 by t-test; n = 20 cells). **(C)** Quantification of projection morphologies for *sec4-8* and *NΔ200-spa2* mutants treated for 4h. Projection morphology was manually characterized as single, double, and irregular projections for at least 100 cells. **(D)** Co-localization of GFP-Sec4 with Spa2-mCherry. WT cells containing GFP-Sec4 and Spa2-mCherry were treated for 2h. Individual and merged images are shown. **(E)** Localization of GFP-Sec4. WT, *NΔ200-*spa2, and *msb3Δ msb4Δ* cells containing GFP-Sec4 were treated with α-factor for 2h. **(F)** Quantification of GFP-Sec4 polarization in mutant and wild-type strains. Cells were treated for 2h with α-factor. The *msb3Δ msb4Δ* cells showed significantly wider polarization (***, p < 0.001; n = 20 cells) as well as more focal localization as measured by fluorescence intensity ratio between projection tip and cytoplasm (*, p < 0.05 by t-test; n = 20 cells) compared to wild-type.

Given that Msb3/4 act as GAPs for Sec4, we hypothesized that Sec4 is downstream of Msb3/4 in this polarization pathway, and that a *sec4* loss-of-function mutant should exhibit morphological defects during mating polarization. We observed that temperature-sensitive *sec4-8* mutant cells possessed misshapen large single projections at the non-permissive temperature (Fig 5A) after 4h α-factor treatment with significantly broader projections and polarisomes compared to wild-type (Fig 5B). In addition, Fus1-GFP in this mutant showed more diffuse localization and weaker polarization reminiscent of *spa2Δ* cells. Finally, most *sec4-8* cells exhibited a single irregular projection at 4h of pheromone treatment similar to *spa2Δ* (Fig 5C).

If Sec4 is downstream of Spa2 and Msb3/4, then one might expect that Sec4 localization would be disrupted in the *NΔ200-spa2* mutant and the *msb3Δ msb4Δ* mutants. For example, a possible mechanism is that defects in hydrolysis may affect the ability of Sec4 to dock with the membrane altering its distribution [42]. Indeed during budding, *spa2Δ* cells did not localize Sec4 to the bud tip [21]. We constructed GFP-Sec4 under the Sec4 promoter in a centromeric plasmid, and found that Sec4 localized to the polarisome in WT cells (Fig 5D). In *NΔ200-spa2* mutant cells, GFP-Sec4 showed a dispersed and less intense appearance (Fig 5E). We also transformed GFP-Sec4 into the *msb3Δ msb4Δ* mutant and found that the polarization of Sec4 was intermediate between WT and *NΔ200-spa2*. In *msb3Δ msb4Δ* cells (1.1 μm), GFP-Sec4 localization was significantly broader and less polarized than in WT cells (0.87 μm), but less broad than in *NΔ200-spa2* cells (1.4 μm; Fig 5E and 5F).

In summary, we performed genetic analysis which showed defects in polarization caused by a loss-of-function mutation in Sec4. In addition, we observed decreased polarization of Sec4 localization in both *spa2* and *msb3/4* mutants. These data are consistent with the putative positive feedback pathway from Spa2 SHD to Msb3/4 to Sec4.

### Bud6 localization to polarisome depends on Spa2, Msb3/4, and Sec4

The next step was to identify what is downstream of Sec4 in this polarization pathway. One likely candidate is Bud6, which is the third principal member of the polarisome along with Spa2 (Pea2) and Bni1. It stimulates the formation of actin cables by Bni1 [14, 32]. The *bud6Δ* mutants are able to bud, but exhibit fewer actin cables [43]. Importantly, Jin and Amberg [30] demonstrated that during budding Bud6 spatial dynamics were adversely affected in *sec4* mutants with greater than 50% of cells exhibiting mislocalized Bud6 in the *sec4-8* temperature-sensitive mutant. Thus, we wished to test the hypothesis that disruption of the pathway upstream of, or at Sec4 would reduce Bud6 polarization during pheromone treatment.

**Fig 6:**
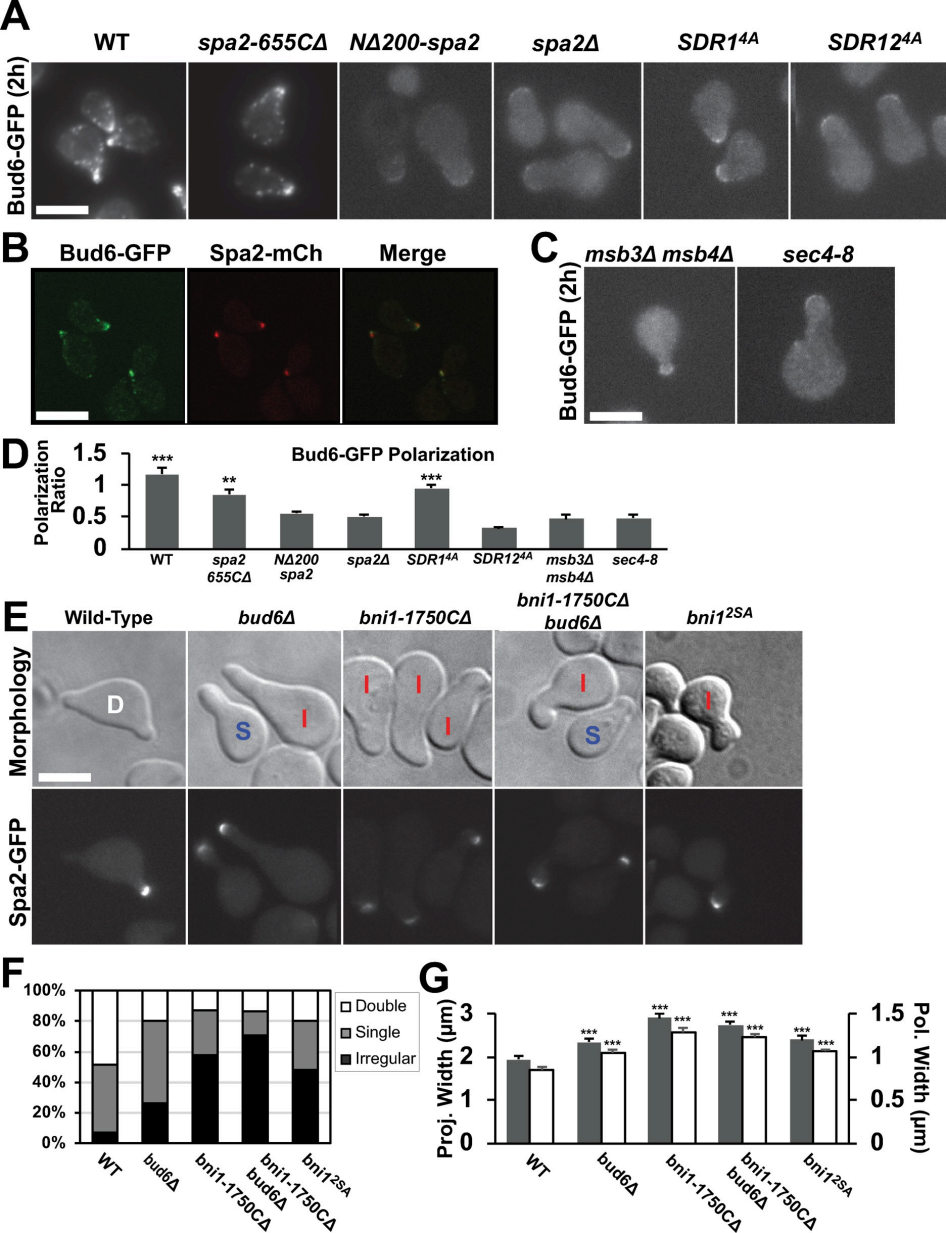
Polarization of Bud6-GFP and morphological defects in *bud6/bni1* mutants. **(A)** Bud6 localization in WT and mutant *spa2* strains. Bud6-GFP containing cells were treated with α-factor for 2h. Bud6 displayed a dispersed, non-focal appearance in the *NΔ200-spa2*, *spa2Δ* and *SDR12*^*4A*^ strains. Scale bars = 5 μm in figure. **(B)** Co-localization of Bud6-GFP with Spa2-mCherry. WT cells containing Bud6-GFP and Spa2-mCherry were treated for 2h with α-factor and then imaged by confocal microscopy. **(C)** Bud6 localization in strains containing mutations in Msb3/4 and Sec4. The double mutant *msb3Δ msb4Δ* and *sec4-8* (at the non-permissive temperature) cells containing Bud6-GFP were treated for 2h with α-factor. **(D)** Quantification of Bud6-GFP polarization in *spa2*, *msb3/4*, and *sec4* mutants. The polarization ratio of fluorescence intensity of the projection tip versus cytoplasm was significantly higher in WT, *SDR1*^*4A*^, and *spa2-655CΔ* compared to *spa2Δ* (**, p < 0.01; ***, p < 0.001 by t-test; n = 20 cells). **(E)** Morphology and polarisome phenotypes in WT, *bud6Δ* and *bni1* mutant cells after 4h α-factor treatment. Many mutants possessed broader mating projections with wider polarisomes that were classified as irregular (I). **(F)** Quantification of projection morphologies for *bud6Δ*, *bni1-1750CΔ*, *bni1-1750CΔ bud6Δ*, and *bni1*^*2SA*^ (*bni1*^*S1819A*, *S1820A*^) mutants treated with α-factor for 4h. Projection morphology was manually characterized as single (S), double (D), and irregular projections (I) for at least 100 cells. **(G)** Quantification of projection and polarisome widths in *bud6* and *bni1* mutant strains after 4h α-factor treatment. In all mutants, both widths were significantly broader than wild-type (***, p < 0.001 by t-test; n = 20 cells).

First we examined Bud6-GFP polarization in WT and *spa2* mutant cells (Fig 6A) to test this hypothesis. In WT cells during the pheromone response, Bud6 primarily localizes to the polarisome (Fig 6B). In *spa2Δ* cells, the focal localization of Bud6-GFP is lost resulting in broader polarization. The *spa2-655CΔ* mutant resembled WT with a punctate Bud6-GFP appearance, whereas the *NΔ200-spa2* and *SDR12*^*4A*^ mutants resembled *spa2Δ*; *SDR1*^*4A*^ possessed an intermediate Bud6-GFP localization (Fig 6A). We quantified the focal polarization by calculating the focal intensity of Bud6 in the polarisome versus the cytoplasm and found significantly higher focal localization of Bud6 in WT and *spa2-655CΔ* compared to *NΔ200-spa2* and *spa2Δ*. As a control for protein expression, we examined the levels of Bud6-GFP in the various mutant strains. By Western blot, we found less than a 15% difference in total Bud6-GFP from wild-type (S5 Fig).

Based on the pathway, one would expect loss-of-function mutations in Msb3/4 and Sec4 would also disrupt Bud6 localization, and so we examined the spatial distribution of Bud6 in *msb3Δ msb4Δ*, and *sec4-8* cells (Fig 6C). We found that Bud6 displayed an abnormal distribution with reduced punctate polarization. Quantification indicated a noticeable reduction in focal polarization of Bud6-GFP at the membrane and a wider distribution (Fig 6D). These data indicate that Spa2 SHD, Msb3/4, and Sec4 are all necessary for proper focal localization of Bud6 at the projection tip consistent with a pathway from SHD to Msb3/4 to Sec4 to Bud6.

### Defective polarization in *bud6Δ* and mutants that disrupt Bud6-Bni1 interaction

To complete the feedback loop, Bud6 stimulates the nucleation phase of Bni1-mediated actin assembly [44] resulting in more actin cables along which more Spa2 is transported to the projection tip according to the model. The absence of focal distribution of Bud6 could partially explain the morphological and polarization defects of *spa2* mutants during mating. Thus, we hypothesized that interfering with this last link in the pathway, the interaction between Bud6 and Bni1, should also perturb polarization. We performed experiments on mutants that disrupt the Bni1-Bud6 interaction to test this hypothesis.

#### Characterizing *bud6Δ* morphology after pheromone treatment

The first mutant we examined was a deletion of Bud6 itself. The *bud6Δ* cells treated with pheromone exhibited morphological defects (Fig 6E). A greater percentage of cells produced a single projection instead of two projections at 4h (Fig 6F), and these projections were broader than WT projections (Fig 6G). Despite the relative absence of cables in *bud6Δ* cells (S6 Fig), Spa2 still polarized to the projection tip (Fig 6E). However, the distribution of Spa2 was significantly broader than the WT puncta (Fig 6G).

#### The *bni1-1750CΔ* mutant displays reduced focal polarization

In addition, we selected a Bni1 mutant that would disrupt the Bni1-Bud6 interaction, and thus break a key link in the proposed positive feedback loop. Bud6 is known to interact with the C-terminal tail of Bni1 [13]. Removing this tail region (*bni1-1750CΔ*) renders actin cable production by Bni1 Bud6-independent [22]. We hypothesized that such a mutant would possess a polarization defect because Bni1 would no longer be positively regulated by the feedback loop component Bud6, and instead would be constitutively active in a feedback-independent fashion, which would disrupt polarization.

In *bni1-1750CΔ* cells, the projection was broader and larger, resembling *spa2Δ* (Fig 6E). The mutant polarisome marked by Spa2-GFP was wider than wild-type (Fig 6E and 6G), and for many cells was off-center similar to *msb3Δ msb4Δ* cells. The projections were predominantly irregular (Fig 6F).

We predicted a similar phenotype in the *bni1-1750CΔ bud6Δ* double mutant because the C-terminal deletion should cause Bni1 to be Bud6-independent, thus mitigating the effects of the *bud6Δ* deletion. Indeed, the cells displayed a high percentage of irregular projections similar to *bni1-1750CΔ* alone, although the projections were slightly thinner in the double mutant (Fig 6E and 6F). As expected, the double mutant produced abundant actin cables despite the absence of Bud6 (S6 Fig).

*The bni1*^*S1819A*, *S1820A*^ *mutant exhibits polarization defects*. One concern is that truncating the C-terminus of Bni1 may affect more than just its interaction with Bud6. The work of Moseley and Goode [14] showed that the region from 1816–1824 in Bni1 was critical for binding the Bud6 C-terminal domain, and that the double mutant *S1819A S1820A* dramatically reduced this interaction in a pull-down assay. Thus, the double mutant would disrupt the Bud6-Bni1 interaction in a loss-of-function manner (compared to the gain-of-function disruption of the positive feedback loop in the Bud6-independent *bni1-1750CΔ* mutant).

We engineered this mutant strain and assessed its morphology after 4h treatment with α-factor. Although the phenotype was less pronounced than *bni1-1750CΔ*, the *bni1*^*S1819A*, *S1820A*^ mutant (*bni1*^*2SA*^) exhibited significant polarization defects with a broader projection (2.4 vs. 1.95 μm) and polarisome (1.1 vs. 0.85 μm) than wild-type (Fig 6E and 6G). Taken together, the above experiments demonstrate that the *bud6* and *bni1* mutants produce significantly broader polarisomes and projections (Fig 6G), and these mutant morphologies provide evidence of the important roles of Bud6 and Bni1 in the proposed polarization pathway.

### Partial restoration of polarization in *NΔ200-spa2* mutant: Gain-of-function experiments

**Fig 7:**
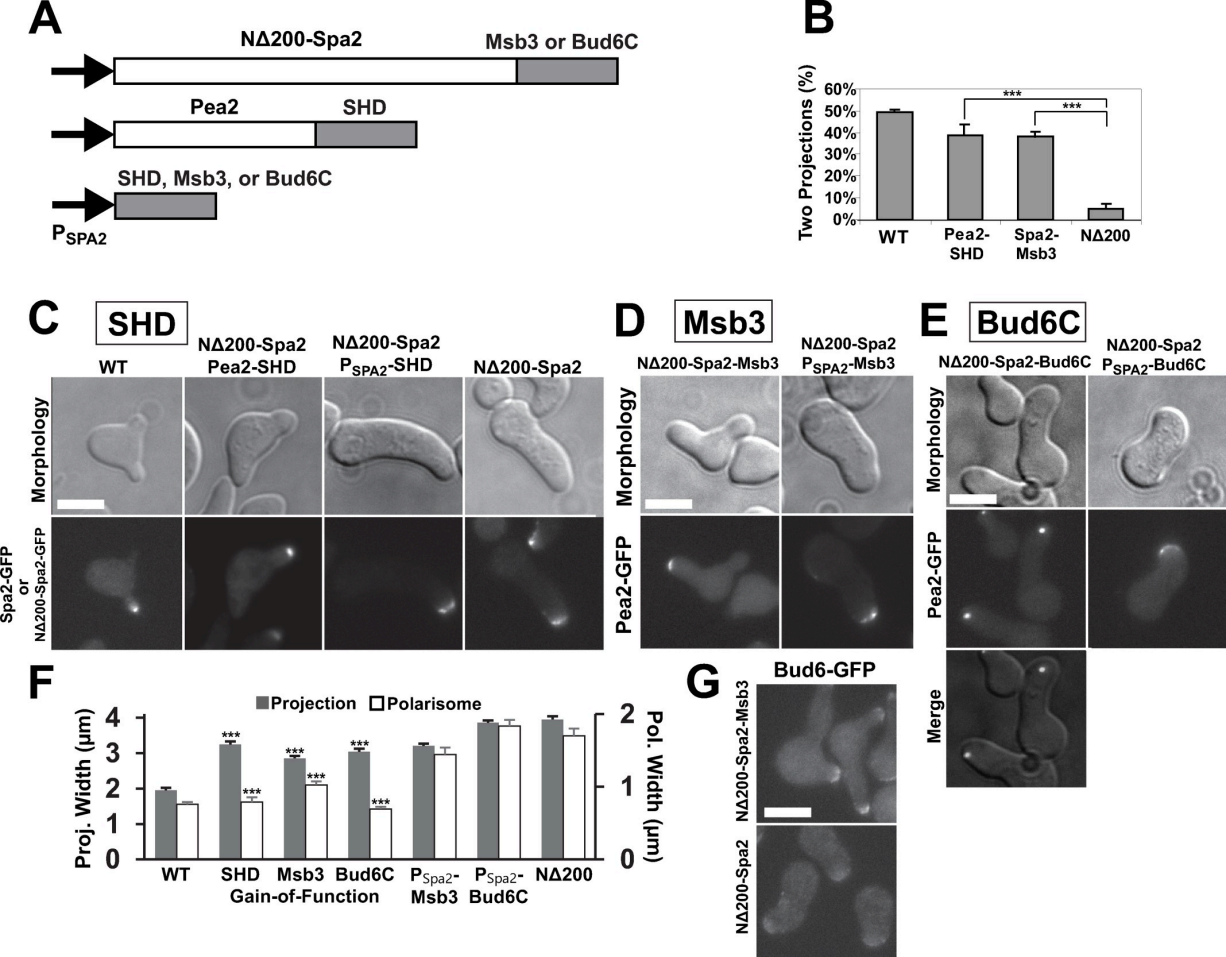
Gain-of-Function experiments in *NΔ200-spa2* background. **(A)** Schematic diagram of experiments. The SHD domain, Msb3, or the C-terminus of Bud6 was fused to the carboxyl end of either N*Δ*200-Spa2 or Pea2, as well as expressed from the *P*_*SPA2*_ promoter. **(B)** Percentage of cells possessing two or more projections. The SHD and Msb3 fusion strains had a significantly higher percentage of double projection cells than *NΔ200-spa2*. Over 100 cells were examined for each strain (***, p < 0.001 by t-test). **(C)** The SHD domain was attached to the C-terminus of Pea2 or expressed from the Spa2 promoter (*P*_*SPA2*_*-SHD*) integrated into the genome (*URA3*). Projection and polarisome (Spa2-GFP) morphologies were imaged after 4h of α-factor treatment. Scale bars = 5 μm in figure. **(D)** Full-length Msb3 was fused on to the end of N*Δ*200-Spa2. As a control, Msb3 was expressed from the Spa2 promoter integrated into the genome. Projection and polarisome (Pea2-GFP) morphologies were imaged after 4h α-factor treatment. **(E)** The C-terminal domain of Bud6 (489 to end) was fused on to the end of NΔ200-Spa2. As a control, the Bud6C alone was expressed from the Spa2 promoter. Projection and polarisome (Pea2-GFP) morphologies were imaged after 4h α-factor treatment. **(F)** Quantification of projection and polarisome widths in gain-of-function mutants. All gain-of-function mutants showed significantly narrower projections and polarisomes compared to *NΔ200-spa2* cells (***, p < 0.001 by t-test; n = 20 cells). **(G)** Bud6-GFP in cells containing NΔ200-Spa2-Msb3 (top) or NΔ200-Spa2 (bottom) treated with α-factor for 4h.

To this point, we employed loss-of-function mutations, which demonstrate necessity; gain-of-function experiments can show sufficiency of a gene function to produce a phenotype. In Fig 7A, we show various protein functional domains (SHD domain, Msb3, and Bud6 C-terminal domain) fused to the C-terminus of either NΔ200-Spa2 or Pea2 in the *NΔ200-spa2* background. We have shown above that in WT cells, the N-terminal domain of Spa2 is necessary for delivering those functional domains to the polarisome. In the *NΔ200-spa2* mutant, the putative positive feedback loop is disrupted. In these gain-of-function experiments, we tested whether we could restore the positive feedback by forced localization of these downstream functional domains to produce more focal polarization, a narrower projection, and more second projections than *NΔ200-spa2* (Fig 7B).

#### Pea2-SHD fusion restores WT polarization in *NΔ200-spa2* mutant

First we attached the N-terminal SHD domain of Spa2 to the C-terminal end of Pea2. We chose Pea2 because Spa2 and Pea2 form a complex from two-hybrid and biochemical data [10, 20] with Spa2 necessary for the stability of Pea2 [20]. Their tight co-localization (S8 Fig) is consistent with the binding data, and in Fig 7, we use both Spa2 and Pea2 to deliver functional domains to the polarisome and as polarisome markers.

The Pea2-SHD construct restored nearly WT polarity in terms of polarisome appearance (Fig 7C). As a control, we expressed the SHD domain alone from the Spa2 promoter (*P*_*Spa2*_*-SHD*) integrated into the genome; this strain had a typical *NΔ200-spa2* phenotype (Fig 7C). The polarisome width was restored to nearly as narrow as wild-type (0.8 μm versus 0.75 μm), while the projection width (3.2 μm) was intermediate between *NΔ200-spa2* (3.95 μm) and wild-type (1.95 μm).

#### NΔ200-Spa2-Msb3 fusion partially restores WT polarization

Second, we attached full-length Msb3 to the C-terminus of NΔ200-Spa2 (Fig 7A) to test whether forced localization of Msb3 could rescue the polarization defects. Many cells formed multiple projections, and the polarisome exhibited an intermediate appearance narrower in width (1.0 μm) than the delocalized polarisome of *NΔ200-spa2* (1.7 μm) but broader than the focal polarisome of WT (Fig 7D); the projection width was also intermediate (2.85 μm) between mutant and wild-type (Fig 7F). Consistent with this partial polarization, Bud6-GFP showed greater polarization in *NΔ200-spa2-MSB3* than in *NΔ200-spa2* alone (Fig 7G). The *P*_*Spa2*_*-MSB3* control was more similar to *NΔ200-spa2* in morphology (3.2 μm) and polarisome (1.4 μm) appearance. Finally, we found that *NΔ200-spa2-MSB3* cells made significantly more double projections than *NΔ200-spa2* cells and a similar number as Pea2-SHD cells, although fewer than WT (Fig 7B).

#### NΔ200-Spa2-Bud6C cells exhibit an internal punctate polarisome

Third, we made a fusion protein between NΔ200-Spa2 and the C-terminus of Bud6 (Bud6C) from 489 to the terminus at 788 corresponding to the functional domain of Bud6 that binds the C-terminus of Bni1 where it helps to recruit actin monomers stimulating actin nucleation by the formin [32, 44] (Fig 7A). The morphology of the *NΔ200-spa2-bud6C* cells was similar to that of *NΔ200-spa2* with a single large projection albeit slightly thinner (Fig 7E). However, visualizing the polarisome with a Pea2-GFP marker showed that it was punctate like WT (Fig 7E) with a width that was slightly narrower (0.7 μm) than WT (0.8 μm). By comparison the *P*_*Spa2*_*-BUD6C* control did not show the punctate polarisome (Fig 7E).

**Fig 8:**
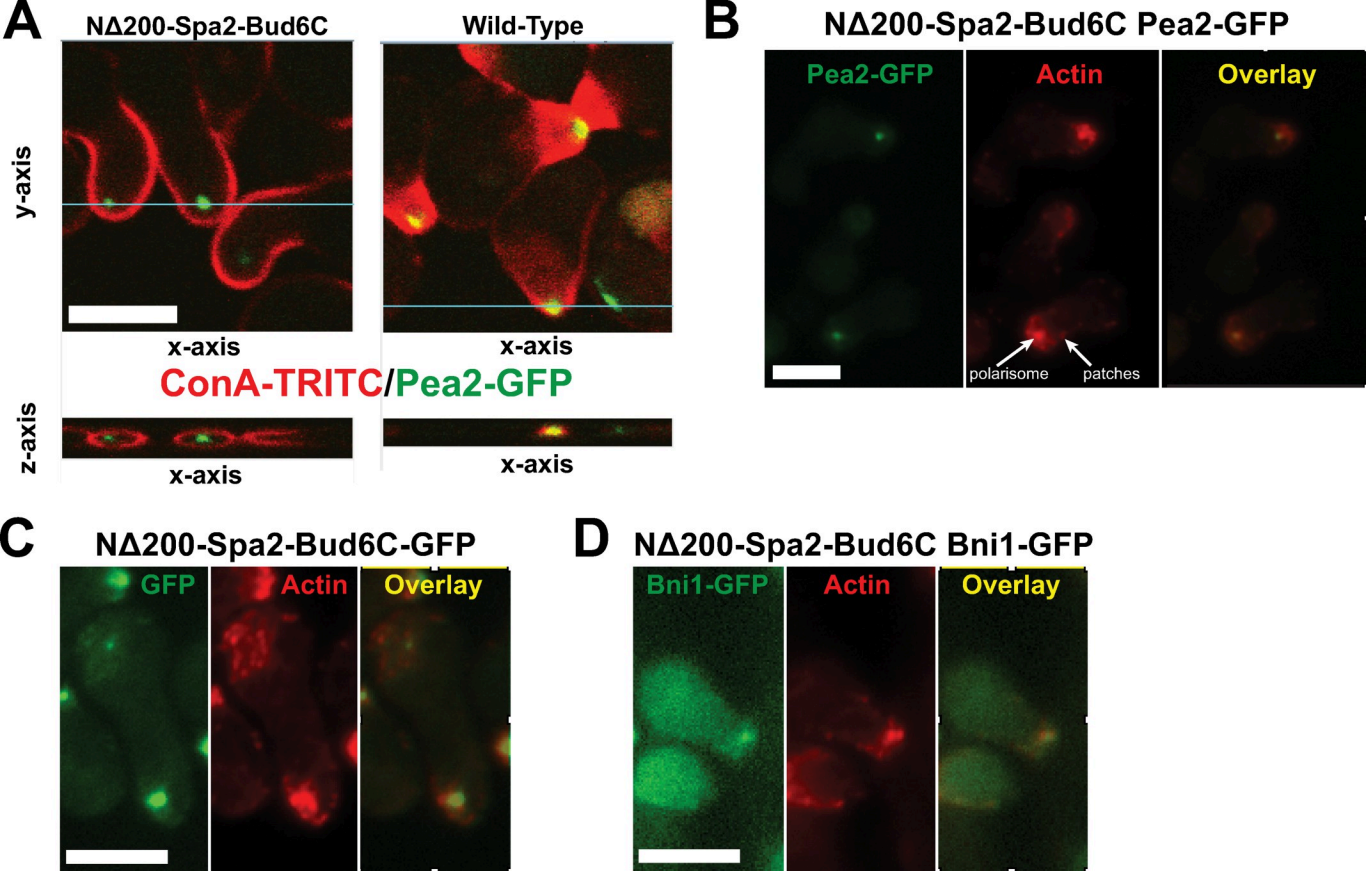
Characterization of polarisome in NΔ200-Spa2-Bud6C gain-of-function mutant cells. **(A)** Images of internal polarisome in cells containing NΔ200-Spa2-Bud6C. NΔ200-Spa2-Bud6C (left) and wild-type (right) cells containing Pea2-GFP were treated with α-factor for 2h, and then ConA-TRITC was added before formaldehyde fixation to stain glycoproteins in the cell wall defining the cell perimeter. A z-stack was constructed with GFP and TRITC image slices taken every 0.3 μm along the *z*-axis, and a 3D reconstruction was created using ImageJ. The *xy*-plane is shown in the top panel, and the *xz*-plane (along the y-coordinate indicated by the blue line in the top panel) is shown in the bottom panel. Some compression along the z-axis in the bottom panel was caused by the coverslip. The polarisome marked by Pea2-GFP was internal to the cell periphery marked by the ConA-TRITC in the NΔ200-Spa2-Bud6C cells. In wild-type cells, the polarisome and cell periphery overlapped. Scale bars = 5 μm in figure. **(B)** NΔ200-Spa2-Bud6C Pea2-GFP, **(C)** NΔ200-Spa2-Bud6C-GFP, and **(D)** NΔ200-Spa2-Bud6C Bni1-GFP cells were treated with α-factor for 2h, fixed, and then stained for actin using rhodamine-phalloidin. The focal distribution of actin (red) co-localized with Pea2-GFP, NΔ200-Spa2-Bud6C-GFP, and Bni1-GFP (green), respectively, in the polarisome (yellow overlay). Arrows in (B) indicate the presence of actin both in the polarisome and in patches at the surface.

However in many cases, the polarisome was not at the surface of the cell, but appeared to be slightly internal to the plasma membrane (Fig 8A). We examined this localization more closely by staining the cell surface with ConA-TRITC and then performing 3D confocal microscopy imaging. In wild-type cells, the polarisome marked by Spa2-GFP overlaps with the ConA-TRITC, whereas in the mutant, it is internal to the cell wall in all three imaging directions including the *z*-axis.

We also examined actin distribution in the NΔ200-Spa2-Bud6C cells and found two patterns: focal actin colocalized with the punctate polarisome (Fig 8B), as seen in WT (Fig 2B), whereas there was a more dispersed actin distribution consisting mainly of actin patches on the surface similar to the *NΔ200-spa2* mutant. The same pattern (punctate internal polarisome along with surface patches) was observed from the NΔ200-Spa2-Bud6C fusion protein directly tagged with GFP (Fig 8C). Similarly, markers upstream of Bud6 (i.e. Msb3-GFP and GFP-Sec4) obviated by the Bud6C forced localization showed the dispersed pattern expected of *NΔ200-spa2* (S7 Fig). These data suggest that the Bud6 C-terminal domain is sufficient when properly localized to create a punctate polarisome, which is able to synthesize actin. Consistent with these data, in *NΔ200-spa2-bud6C* cells, we were able to observe Bni1-GFP co-localized with the internalized polarisome (Fig 8D).

In summary, we were able to partially circumvent the disrupted positive feedback loop in the *NΔ200-spa2* mutant by re-creating the loop through forced localization of SHD, Msb3, and Bud6C to the polarisome. The width of both the polarisome and the projection was significantly narrower in all three gain-of-function mutants compared to the *NΔ200-spa2* mutant background. The NΔ200-Spa2-Bud6C cells showed a striking phenotype in which the polarisome was as narrow as wild-type but in an internal location.

### Reduced polarisome stochasticity in gain-of-function strain

**Fig 9:**
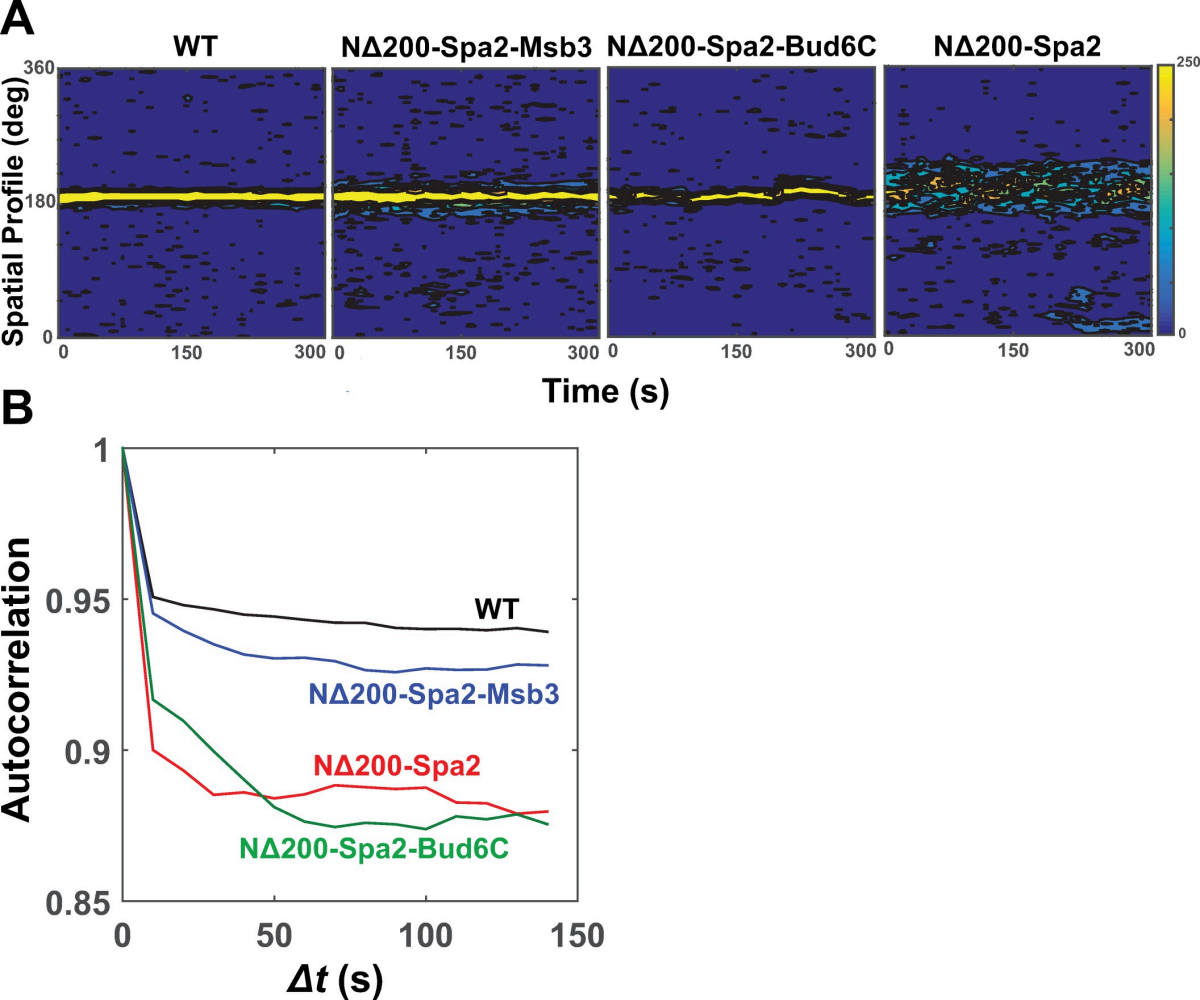
Polarisome dynamics in gain-of-function mutants. **(A)** WT, NΔ200-Spa2-Msb3, NΔ200-Spa2-Bud6C, and NΔ200-Spa2 cells containing Pea2-GFP were imaged for 300s at 10s intervals. Normalized fluorescence intensity (color bar) was plotted as a function of time (*x*-axis) and spatial position (*y*-axis). **(B)** The autocorrelation was calculated from each polarisome intensity plot and then averaged over 5 experiments for each strain.

Both the *spa2Δ* and *NΔ200-spa2* strains exhibited highly random movement of the polarisome mini-clusters compared to the more stationary wild-type polarisome. We wished to examine whether the gain-of-function alterations in the *NΔ200-spa2* background suppressed this stochasticity. We performed time-lapse imaging (10s intervals) of the polarisome in WT, *NΔ200-spa2-msb3*, *NΔ200-spa2-bud6C*, and *NΔ200-spa2* strains labeled with Pea2-GFP (S1–S4 Videos). The spatial dynamics revealed a relatively static, punctate polarisome for WT, whereas the polarisome was broader and dynamic in *NΔ200-spa2* (Fig 9A), consistent with past data [16]. The NΔ200-Spa2-Msb3 cells possessed an intermediate phenotype that more closely resembled WT, while the NΔ200-Spa2-Bud6C cells contained the internal punctate polarisome that was much more mobile than WT (Fig 9A).

Calculation of the fluorescence autocorrelation revealed the highest values in WT and lowest in *NΔ200-spa2*, reflecting their relative polarisome mobilities (Fig 9B). The NΔ200-Spa2-Msb3 cells exhibited an intermediate autocorrelation, whereas autocorrelation in NΔ200-Spa2-Bud6C cells was similar to *NΔ200-spa2* because of the dynamic behavior of the polarisome (Fig 9A). Thus, NΔ200-Spa2-Msb3 conferred a narrower projection morphology, and a narrower, less stochastic polarisome compared to *NΔ200-spa2*, providing evidence that forced localization of Msb3 could partially suppress three key phenotypes. The stochastic nature of the NΔ200-Spa2-Bud6C internal polarisome suggests that the positive feedback pathway may act through other mechanisms other than Bud6 to stabilize polarisome motion. One possibility is that interaction with the membrane and membrane-associated proteins could stabilize the motion.

## Discussion

**Fig 10:**
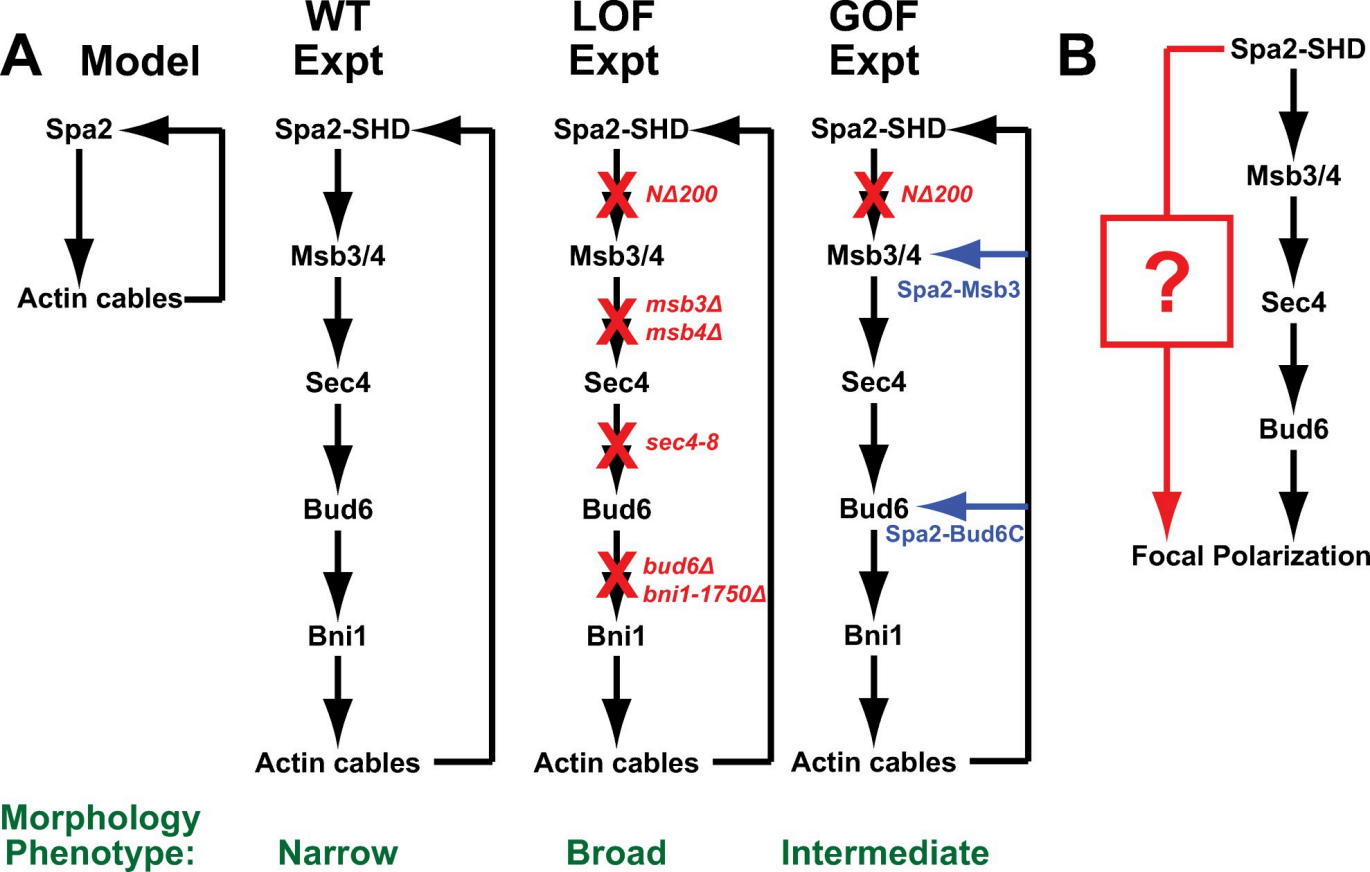
Schematic diagrams of pathways involving Spa2 SHD. **(A)** Arrow diagram of positive feedback loop mediated by Spa2 SHD domain as predicted by the computational model and then delineated by experimental data. In loss-of-function (LOF) experiments, the positive feedback loop is disrupted at different points in the pathway resulting in broader morphology. In gain-of-function (GOF) experiments, the *NΔ200-spa2* mutant is partially suppressed by re-creating the positive feedback loop at a different position in the pathway resulting in intermediate morphology phenotype. **(B)** The proposed existence of a parallel pathway (red) depending on the Spa2 SHD domain that also contributes to focal polarization. This additional pathway could explain the partial loss-of-function and partial gain-of-function phenotypes when the Msb3/4-Sec4-Bud6 pathway (black) is mutated.

In summary, motivated by mathematical modeling and computer simulations, we have characterized and collected data consistent with a novel positive feedback loop involving the Spa2 SHD domain that stimulates Sec4-mediated transport of Bud6 to the polarisome and contributes to focal polarization. Breaking the positive feedback loop at any point results in a loss of focal polarization. We were able to demonstrate significant defects in the mating projection and polarisome morphologies caused by loss-of-function mutations in Msb3/4, Sec4, and Bud6. Gain-of-function experiments partially restored the positive feedback loop in an *NΔ200-spa2* background using “downstream” components (e.g. Msb3 and Bud6C) targeted to the polarisome without restoring localization of putative upstream components. Strikingly, attaching the functional domain of Bud6 to NΔ200-Spa2 was able to re-form a punctate polarisome, albeit in an unusual position slightly internal to the cell membrane. In this manner, we traced the pathway from Spa2 to Bud6 that contributes to proper polarization during the pheromone response (Fig 10A).

Our work was motivated and guided by computer simulations. The simulations previously predicted the presence of two positive feedback loops (Loop 1 and Loop 2) necessary for focal polarization [16]. Loop 2 was novel involving the actin cytoskeleton, which would direct Spa2 to the polarisome (S8 Fig) where it would increase the number of actin cables (Fig 10A). More specifically, the critical reactions occur in the polarisome which is tethered to the membrane through interactions with membrane-bound species such as Cdc42. There, Bni1 synthesizes actin cables directing secretory vesicles containing Spa2 to the polarisome. As shown by experiments, the N-terminus of Spa2 recruits Msb3/4 to the polarisome where we propose it promotes Sec4-mediated transport of secretory vesicles containing Bud6 to the polarisome, further stimulating Bni1 activity.

There was a more dramatic defect in polarization and morphology when Loop 2 was disrupted compared to Loop 1. The *NΔ200-spa2* mutant exhibited a phenotype similar to the *spa2Δ* full deletion mutant. Indeed, Arkowitz and Lowe [15] demonstrated only a modest decline in mating efficiency and fraction polarized in the *spa2-655CΔ* and *spa2-1074CΔ* C-terminal deletions, which was consistent with our data showing modest decreases in Spa2, Fus1 and Bud6 polarization in these mutants compared to wild-type. Thus, although our modeling indicated that the Spa2 C-terminal interaction with Bni1 (Loop 1) could be important for polarisome formation, and this interaction has been characterized in the literature [22], our experimental results showed that Loop 1 plays a minor role in focal polarization especially compared to Loop 2 suggesting that the direct Spa2-Bni1 interaction may be dispensable for mating.

Spa2 plays a less critical role in budding i.e. the morphology of the bud is relatively normal in a *spa2Δ* mutant. One difference between budding and mating is that bud formation does not require sustained focal polarization; indeed, the rounder bud shape observed in *spa2Δ* is the consequence of more dispersed, spreading polarization. Likewise the loss-of-function mutations in Msb3/4 and Bud6 do not have pronounced budding morphological defects [14, 24, 30].

Previously, Moseley and Goode predicted that Bud6 might be part of a positive feedback loop during budding [14, 44]. They proposed that Bud6 enhanced Bni1 actin cable formation that delivered more Bud6 to the bud tip. We have confirmed this prediction in part by demonstrating that Bud6 is part of a positive feedback loop that is required for focal polarization during mating. Interestingly, a recent paper [45] demonstrates that Boi1/2 direct Bni1 and Bud6 to sites of exocytosis, suggesting that additional factors may play a role in this feedback loop.

Importantly, this work on mating complements the research on budding for this pathway. First, we provided supporting evidence for the pathway ordering from Spa2 SHD to Msb3/4 [24], from Msb3/4 to Sec4 [40], and from Sec4 to Bud6 [30], and from Bud6 to Bni1 [33, 44] that was originally proposed in budding. Second, we were able to collect data consistent with the hypothesis that the pathway forms a positive feedback loop that contributes to focal polarization during mating.

SHD domains are evolutionarily conserved, playing important roles in focal polarization and secretion in higher eukaryotic pathways. For example, SHD proteins are involved in the assembly of the cytoskeletal matrix at active zones (CAZ) where neurotransmitter is released into the mammalian synapse. More specifically, the protein Piccolo (which is a core component of the CAZ) contains an SHD domain, which has been shown to bind GIT1, a member of the GIT family of GTPase-activating protein. Similar to Spa2, Piccolo is a large scaffold protein with an SHD domain, interacts with actin cytoskeleton regulators, and is transported in vesicles to a polarization region. Similar to Msb3/4, GIT1 is a GAP for a small G-protein that localizes to a focal region and binds the SHD domain [23].

The irregularly shaped (I) projections observed in the *msb3/4Δ*, *sec4-8*, *bud6Δ*, and *bni1* mutants could arise from re-polarization events that occur close to the previous site of polarization. In other words, the polarisome in the mutants may be more likely to disassemble and then reassemble in a neighboring location (e.g. off-center) resulting in an irregular projection. We plan to make a more detailed characterization of the spatial dynamics of these mutants in the future.

We note that the loss-of-function mutations in Msb3/4, Sec4, or Bud6 failed to fully reproduce the *spa2Δ* phenotype, and that the gain-of-function modifications did not fully restore the WT phenotype. Overall, the *msb3Δ msb4Δ* (2.6 μm projection width, 0.8 μm polarisome width), *sec4-8* (2.75 μm, 1.2 μm), and *bni1*^*S1819A*, *S1820A*^ (2.4 μm, 1.1 μm) mutants all displayed an intermediate phenotype between the narrow wild-type (1.95 μm, 0.85 μm) and the broad *NΔ200-spa2* (3.9 μm, 1.7 μm) morphologies after 4h of α-factor treatment. Likewise, the gain-of-function mutants *NΔ200-spa2-MSB3* (2.9 μm, 1.0 μm) and *NΔ200-spa2-bud6C* (3.0 μm, 0.7 μm) also exhibited intermediate morphological phenotypes between wild-type and *NΔ200-spa2* with the exception of the more focal polarisome observed in *NΔ200-spa2-bud6C*. In addition, the effect of the upstream loss-of-function mutations seemed more pronounced on Bud6 polarization than on Spa2 polarization. Finally, a note of caution when interpreting gain-of-function variants when the proteins involved are pleiotropic, e.g. Msb3 is involved in multiple pathways [46]; however, it is striking how all three gain-of-function constructs restored at least partial wild-type polarisome polarization in the *NΔ200-spa2* background.

The intermediate nature of the mutant phenotypes suggests the existence of further uncharacterized pathways mediated by the N-terminal domain of Spa2 necessary for wild-type focal polarization; the proposed Loop 2 pathway is not sufficient. One possibility is that the Spa2 SHD domain binds additional proteins along with Msb3/4 that contribute to polarization. For example, Sheu et al. showed by two-hybrid assay that the SHD domain also binds the MEKs of the cell wall integrity pathway [21]. We plan to investigate this hypothesized additional pathway for focal polarization (Fig 10B).

On the surface, it is puzzling that GAP proteins Msb3 and Msb4 stimulate secretion by catalyzing the deactivation of Sec4. However, as pointed out by Novick and colleagues, “the ability of Sec4 to cycle between the GTP- and GDP-bound forms rather than the absolute levels of the GTP-bound form that is critical for function” [28]. One key role of Sec4 is mediating the docking of exocytic vesicles with the exocyst [47, 48]. Importantly, Donovan and Bretscher recently demonstrated a dramatic increase in aborted secretory vesicle fusion events with the plasma membrane in the *sec4*^*Q79L*^ GTP-hydrolysis mutant [42]. Similarly, others have demonstrated in yeast that GAPs and guanosine dissociation inhibitors (GDIs, which maintain G-proteins in the GDP inactive state) can stimulate Cdc42 recycling which is necessary for proper polarization [49], while in mammalian cells Soldati et al. showed that the rab3A-GDI is an important recycling factor for the transport protein rab9 [50].

Recent work by Xie at al. [51] examined the functional significance of a previously uncharacterized member of the polarisome, Aip5, which promotes actin assembly by Bni1. Localization of Aip5 to the bud tip depends on the C-terminus of Spa2. Under stress conditions (e.g. energy depletion) Aip5 formed cytoplasmic condensates that are regulated by Spa2 via a novel liquid-liquid phase separation mechanism. The role of Aip5 during mating remains to be determined although we note that the Spa2 C-terminus can be deleted with only a modest effect on mating-induced focal polarization, and that our work was primarily concerned with the functional roles of the N-terminus of Spa2.

The phase separation model proposed by Xie at al. [51] highlights an alternative process to drive polarisome formation. Both our work and the paper of Dünkler et al. [35] provide evidence for a positive feedback loop that underlies focal polarization. Other possible mechanisms include clustering and cooperativity [52], as well as stochastic effects [16, 53]. Although we did not do so, it should be possible to model the polarisome as a large soluble complex that may form in a cooperative fashion through the interactions of numerous subunits and filaments that are localized to a specific location [54]. These mechanisms are not mutually exclusive, and some combination is likely to contribute to polarisome assembly. Indeed it has been shown that positive feedback in combination with cooperativity [55] or stochastic effects [16, 56] can generate enhanced polarization.

Finally, Dünkler et al. showed that an interaction between Pea2 and the type V Myosin Myo2 is necessary for proper polarization of the polarisome during budding [35]. Disrupting this interaction by a single point mutation in the Myo2 cargo domain resulted in a broader polarisome, and round versus elongated buds. The authors provided evidence of a positive feedback pathway involved in this polarization including roles for Msb3/4, Bud6, Bni1, and Spa2. Disrupting the pathway produced defects in polarization and bud morphology analogous to the alterations we observed during mating. Interestingly, they extended their findings to mating, and demonstrated delayed polarisome formation and reduced mating efficiency. Taken together these results suggest that proper polarization during budding requires actin, myosin, and polarisome components acting in concert to localize the Pea2–Myo2 complex towards the center of the polarisome, helping to concentrate Bni1 and Bud6 at the bud tip where they produce more actin cables resulting in a positive feedback loop. These conclusions are consistent with and complementary to our work in terms of the positive feedback architecture, pathway components, and phenotype (focal polarization). In terms of differences, our work exclusively examined mating, whereas Dünkler et al. concentrated primarily on budding. In addition, we characterized the interaction between the Spa2-SHD and Msb3/4 versus their focus on the Pea2-Myo2 interaction. Both interactions assisted in recruiting secretory transport components to the polarisome. Together the two studies add to our understanding of polarisome polarization during budding and mating.

One example of the complementarity is that the Dünkler et al. paper proposes a plausible handover mechanism in which Myo2 first detaches from Sec4 (via Msb3/4 catalyzed GTP hydrolysis) before binding to Pea2 in order to be concentrated at the polarisome. The Myo2 can then act together with newly synthesized actin cables to pull vesicles and other components toward the center of the polarisome resulting in more focal localization. This explanation ties together how the Msb3/4-Spa2 interaction described in this work can combine with the Pea2-Myo2 interaction described in Dunkler et al. in the context of the positive feedback pathway outlined in both papers to promote focal polarisome formation.

We updated our model of the polarisome [16] to include elements of this new pathway and especially the role of Spa2 to recruit Bud6 to stimulate the formation of more actin cables (S1 Text). Yeast has been extensively modeled as a cell polarity system. Much of the mathematical modeling has been devoted to the role of Cdc42, the master regulator of polarization, during budding [56–59]. In addition, we and others have modeled mating-induced polarization [55, 60, 61] which is different from budding because the mating projection can track an external chemical pheromone gradient via the heterotrimeric G-protein dynamics, and because of the enhanced polarization (i.e. mating projection is narrower than the bud). A key element of the focal polarization during pheromone treatment is the polarisome which lies downstream of Cdc42 and is one of the most punctate structures in the mating projection. Our previous efforts represented the first attempt to model the polarisome including the role of stochastic spatial dynamics during mating [16]. In addition, recent work has demonstrated that tight polarization can also contribute to directional persistence, another important feature of mating polarization [62].

In the future, we plan to include endocytosis and exocytosis more explicitly in our models of focal polarization during yeast mating. The *spa2Δ* cells may possess defects in endocytosis that contribute to the larger and broader projection; modeling actin patches along with actin cables would address this question. A more mechanistic description of the polarized transport and secretion pathway would include elements such as the exocyst and the myosin V homolog Myo2 [48].

## Materials and methods

### Yeast strains and cell culture

All yeast strains were derivatives of W303-1a and contained the *bar1Δ* deletion so that α-factor was not degraded during the longer pheromone treatments. Genetic techniques were performed according to standard methods [63]. Complete strain details are presented in S5 Table. Cells were cultured in YPD media (yeast extract-peptone-dextrose media) unless otherwise indicated. Gene deletions, GFP-tagging, and gene-fusions were constructed by genomic integration using vectors amplified and targeted by PCR primers [64].

### Microscopy

Pheromone-induced cells were treated with 1 μM α-factor for the designated time prior to imaging. For static images, cells were fixed in 3.7% formaldehyde for 10 minutes and then mounted on slides using Vectashield (Vector Labs). Bright-field and fluorescence imaging were performed using a Nikon Eclipse TE-2000 system with a 60x (NA = 1.4) objective. Confocal imaging was performed using an Olympus Fluoview 1000 Spectral confocal microscope with a 60x (NA = 1.4) objective. For actin visualization, cells were stained with rhodamine-phalloidin (Invitrogen) after formaldehyde fixation. For cell wall visualization, cells were treated with ConA-TRITC (Molecular Probes) at 10 μg/ml.

Time-lapse imaging was performed on the Olympus Spectral confocal microscope with cells adhered to glass slides using Concanavalin A and maintained in YPD media containing 1 μM α-factor at room temperature.

### Image analysis

Images were initially processed by ImageJ [65] and then analyzed using custom software written in Matlab [16]. Projection number and morphology were determined manually.

Autocorrelation analysis of polarisome dynamics was performed in Matlab using the autocorr2d.m function. Data were from time-lapse confocal images of a polarisome marker taken every 10s for 300s. Threshold filters were used to remove imaging noise.

## Supporting information

S1 Fig Autocorrelation plots for simulations of spa2 mutants. The *y*-axis represents autocorrelation in time *G(0*,*Δt)*, and the *x*-axis is the time step *Δt* in seconds. Simulations for WT (blue, wild-type), spa2-L1 (green, only first positive feedback loop), spa2-L2 (orange, only second positive feedback loop), and spa2Δ (red) are shown. The solid lines indicate average of 100 simulations, and the dotted lines represent sample trajectories. Simulations are from two different parameter sets. Left is *B*_*fb*_*/B*_*on*_ and *K*_*m*_ low, and right is *B*_*fb*_*/B*_*on*_ and *K*_*m*_ high. In both cases, the trend is that autocorrelation decreases from WT to spa2-L1 to spa2-L2 to spa2Δ.
(PDF)

S2 Fig Images of polarisome marker proteins in *spa2* deletion mutant backgrounds. In WT, *spa2-1074CΔ*, *spa2-655CΔ*, *spa2-511CΔ*, and *spa2Δ* backgrounds, the C-terminus of Spa2, Pea2, Myo2, and Sec3 were labeled with GFP. Cells were treated with α-factor for 2h, and imaged by confocal microscopy. The *spa2-511CΔ* and *spa2Δ* strains did not produce detectable Spa2-GFP or Pea2-GFP. ND indicates not determined. Scale bar = 5 μm.
(PDF)

S3 Fig Fluorescent images of wild-type and *spa2* mutant cells containing Bni1-GFP treated with α-factor for 2h. The width of Bni1-GFP polarization (below) was measured in microns and compared to wild-type (***, p < 0.001; **, p < 0.01; n = 20 cells). Scale bar = 5 μm.
(PDF)

S4 Fig Localization of Msb/Gyp proteins in spa2 mutant backgrounds. Gyp2, Msb3, Msb4, and Gyp5 were tagged with GFP in wild-type (WT), *SDR1*^*4A*^, *SDR2*^*4A*^, *SDR12*^*4A*^, and *spa2Δ* strains. Imaging was performed after 2h pheromone treatment. Some of these data (Msb3-GFP and Msb4-GFP) are reproduced in Fig 3B of the main text. Gyp2-GFP and Gyp5-GFP show some polarization in the *SDR2*^*4A*^ strain, whereas Msb3-GFP and Msb4-GFP do not. Gyp5-GFP also shows some polarization in the *SDR12*^*4A*^ and *spa2Δ* strains, whereas Msb3-GFP and Msb4-GFP do not. Scale bar = 5 μm.
(PDF)

S5 Fig Western blot of Bud6-GFP in *spa2*, *msb3/4*, and *sec4* mutant strains. Cells were treated with α-factor for 2 hours, and then cell extracts were prepared. Western blots were performed with anti-GFP and anti-α-tubulin antibodies, and band fluorescence was imaged using the LI-COR Odyssey system. The bar graph underneath shows quantitization of Bud6-GFP band relative to the α-tubulin band. The mutant GFP/Tubulin ratios were normalized to the wild-type ratio. The mean and standard deviation from three trials are shown. None of the mutant ratios were significantly different from wild-type by t-test.
(PDF)

S6 Fig Actin cytoskeleton in *bud6* and *bni1* mutant strains. Cells were treated with α-factor for 2 hours, and then stained with rhodamine-phalloidin. Scale bar = 5 μm.
(PDF)

S7 Fig Msb3-GFP and GFP-Sec4 in NΔ200-Spa2-Bud6C cells. Msb3-GFP and GFP-Sec4 were imaged in NΔ200-Spa2-Bud6C cells after treatment with α-factor for 2h (third column) and then imaged. Scale bar = 5 μm. For comparison Msb3-GFP and GFP-Sec4 polarization in wild-type (WT, column 1) and spa2 (column 2) strains are also shown. Although the polarisome is punctate in the gain-of-function NΔ200-Spa2-Bud6C cells, the upstream components Msb3-GFP and GFP-Sec4 exhibit the more dispersed appearance found in *spa2* cells.
(PDF)

S8 Fig Effect of latrunculin A (LatA) on Spa2 polarization. **(A)** Wild-type Spa2-GFP cells were treated with 1 μM α-factor for 90 min, and then exposed to 50 μM latrunculin A for 15 min. Typical cells before (t = 0) and after LatA (t = 15m) addition are shown (fluorescence and merged fluorescence (green) and bright-field). Note that it is not the same cells in the field of view, but rather representative cells from a time-course experiment. **(B)** Co-localization of Spa2-mCherry with Myo2-GFP, Pea2-GFP, and GFP-Sec4 after LatA exposure and washout. Cells were treated with α-factor for 1h, and then exposed to LatA for 15m (+LatA), followed by removal of LatA for 15m (-LatA) and continued α-factor treatment throughout. Representative cells are shown in this time-course experiment from the GFP and mCherry channels as well as the merged images. **(C)** Time-lapse experiment in which Myo2-GFP/Spa2-mCherry cells were continually treated with 1 μM α-factor. After 1h initial treatment, LatA was introduced into the culture chamber for 15m (+LatA), and then washed out with α-factor containing YPD, followed by imaging for another 15m (-LatA). The same cells were followed in the experiment with the cell periphery outlined in the dashed white lines. GFP, mCherry, and merged images are shown. In all the experiments, Spa2 polarizes to the polarisome during initial mating factor response, de-polarizes during LatA treatment, and then re-polarizes to polarisome after LatA is removed. Myo2, Pea2, and Sec4 co-localize with Spa2 during these dynamics. Scale bar = 5 μm.
(PDF)

S9 Fig Bud6 spatial dynamics in Bud6 model simulations. Left: time-series visualization of spatial localization of Bud6 on the membrane (in degrees) on the *y*-axis and time on the *x*-axis. Molecule numbers are color-coded by the color bar. Right upper: Numbers of Bud6 on the membrane as a function of time for a sample simulation. Right lower: snapshot of spatial profile of Bud6 on the membrane at a sample time point after polarization.
(PDF)

S10 Fig Spa2 spatial dynamics in Bud6 model simulations. Left: time-series visualization of spatial localization of Spa2 on the membrane (in degrees) on the *y*-axis and time on the *x*-axis. Molecule numbers are color-coded by the color bar. Right upper: Numbers of Spa2 on the membrane as a function of time for a sample simulation. Right lower: snapshot of spatial profile of Spa2 on the membrane at a sample time point after polarization.
(PDF)

S11 Fig Results from a parameter sweep of the default polarisome model. Parameter sweep has the *K*_*m*_ values on the *x*-axis and the ratio of *B*_*fb*_*/B*_*on*_ on the *y*-axis. (A) Regions of parameter space with tight polarization and accurate tracking. Red indicates areas where the width is sufficiently polarized (FWHM < 18 degrees); blue indicates areas where tracking probability is greater than 70%; Purple indicates areas where both conditions are satisfied. (B) Width of the polarisome measured as the FWHM as a best-fit Gaussian. (C) Absolute uncertainty in the width measurement. (D) Probability of successfully tracking a moving input. (E) Absolute uncertainty in the tracking probability.
(PDF)

S12 Fig Results from a parameter sweep of the Bud6 model for the Bud6-related parameters. Parameter sweep has the *B6*_*fb*_ parameter values on the *x*-axis and the *B6*_*on*_ parameter values on the *y*-axis. (A) Regions of parameter space with tight polarization and accurate tracking. Red indicates areas where the width is sufficiently polarized (FWHM < 18 degrees); blue indicates areas where tracking probability is greater than 70%; Purple indicates areas where both conditions are satisfied. (B) Width of the polarisome measured as the FWHM as a best-fit Gaussian. (C) Absolute uncertainty in the width measurement. (D) Probability of successfully tracking a moving input. (E) Absolute uncertainty in the tracking probability. (F) Number of Bud6 molecules on the membrane at steady-state (Bud6-SS). (G) Absolute uncertainty in Bud6-SS numbers.
(PDF)

S1 Table Alpha-factor halo assay on wild-type and mutant strains. (PDF)

S2 Table Biochemical species and initial conditions for the polarisome models. (PDF)

S3 Table Reactions and reaction rates for the original and Bud6 polarisome models. (PDF)

S4 Table Parameters and parameter values for the original and Bud6 polarisome models. (PDF)

S5 Table Yeast strains used in this study. (PDF)

S1 Text Original and modified (Bud6) model of yeast polarisome during pheromone response. (PDF)

S1 Video Time-lapse imaging of Pea2-GFP (polarisome) in wild-type cells treated with 1 μM α-factor for 2h and then imaged for 300s at 10s intervals. (ZIP)

S2 Video Time-lapse imaging of Pea2-GFP (polarisome) in NΔ200-Spa2-Msb3 cells treated with 1 μM α-factor for 2h and then imaged for 300s at 10s intervals. (ZIP)

S3 Video Time-lapse imaging of Pea2-GFP (polarisome) in NΔ200-Spa2-Bud6C cells treated with 1 μM α-factor for 2h and then imaged for 300s at 10s intervals. (ZIP)

S4 Video. Time-lapse imaging of Pea2-GFP (polarisome) in NΔ200-Spa2 cells treated with 1 μM α-factor for 2h and then imaged for 300s at 10s intervals (ZIP)

S1 Raw images (PDF)

S1 Data (XLSX)

## Abbreviations

SHD: Spa2 Homology Domain
SDR: Spa2 Direct Repeat
