# Supplementary material for: A positive feedback loop involving the Spa2 SHD domain contributes to focal polarization
Source: PLoS One. 2022 Feb 8;17(2):e0263347. doi: 10.1371/journal.pone.0263347 (PMC8824340; doi:10.1371/journal.pone.0263347)
Supplement: S1 Fig — The y-axis represents autocorrelation in time G(0,Δt), and the x-axis is the time step Δt in seconds. Simulations for WT (blue, wild-type), spa2-L1 (green, only first positive feedback loop), spa2-L2 (orange, only second positive feedback loop), and spa2Δ (red) are shown. The solid lines indicate average of 100 simulations, and the dotted lines represent sample trajectories. Simulations are from two different parameter sets. Left is Bfb/Bon and Km low, and right is Bfb/Bon and Km high. In both cases, the trend is that autocorrelation decreases from WT to spa2-L1 to spa2-L2 to spa2Δ. (PDF) [file pone.0263347.s001.pdf]

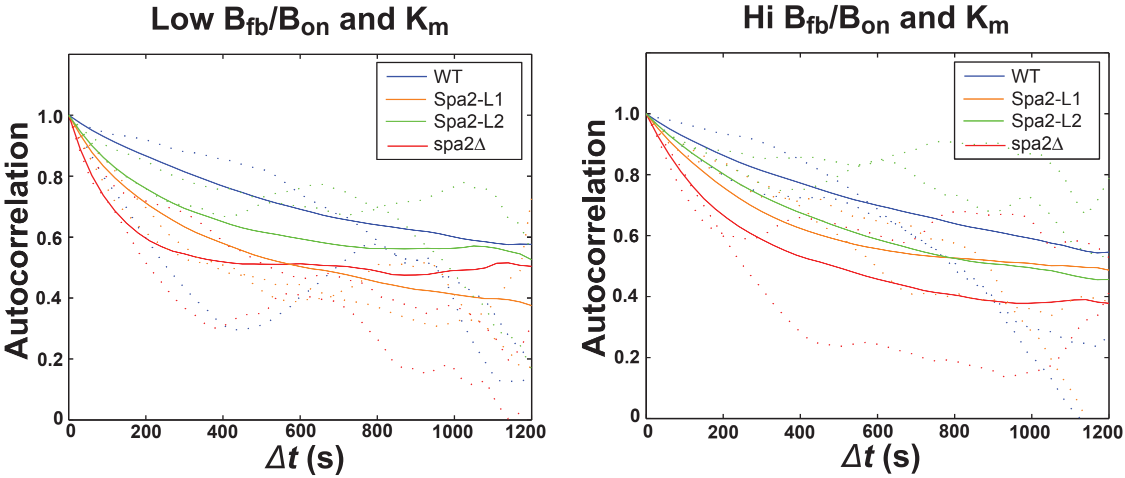

S1 Fig. Autocorrelation plots for simulations of *spa2* mutants. The *y*-axis represents autocorrelation in time $G(0,\Delta t)$, and the *x*-axis is the time step Δt in seconds. Simulations for WT (blue, wild-type), spa2-L1 (green, only first positive feedback loop), spa2-L2 (orange, only second positive feedback loop), and spa2Δ (red) are shown. The solid lines indicate average of 100 simulations, and the dotted lines represent sample trajectories. Simulations are from two different parameter sets. Left is B_{fb}/B_{on} and K_m low, and right is B_{fb}/B_{on} and K_m high. In both cases, the trend is that autocorrelation decreases from WT to spa2-L1 to spa2-L2 to spa2Δ.
