# Supplementary material for: A positive feedback loop involving the Spa2 SHD domain contributes to focal polarization
Source: PLoS One. 2022 Feb 8;17(2):e0263347. doi: 10.1371/journal.pone.0263347 (PMC8824340; doi:10.1371/journal.pone.0263347)
Supplement: S2 Fig — In WT, spa2-1074CΔ, spa2-655CΔ, spa2-511CΔ, and spa2Δ backgrounds, the C-terminus of Spa2, Pea2, Myo2, and Sec3 were labeled with GFP. Cells were treated with α-factor for 2h, and imaged by confocal microscopy. The spa2-511CΔ and spa2Δ strains did not produce detectable Spa2-GFP or Pea2-GFP. ND indicates not determined. Scale bar = 5 μm. (PDF) [file pone.0263347.s002.pdf]

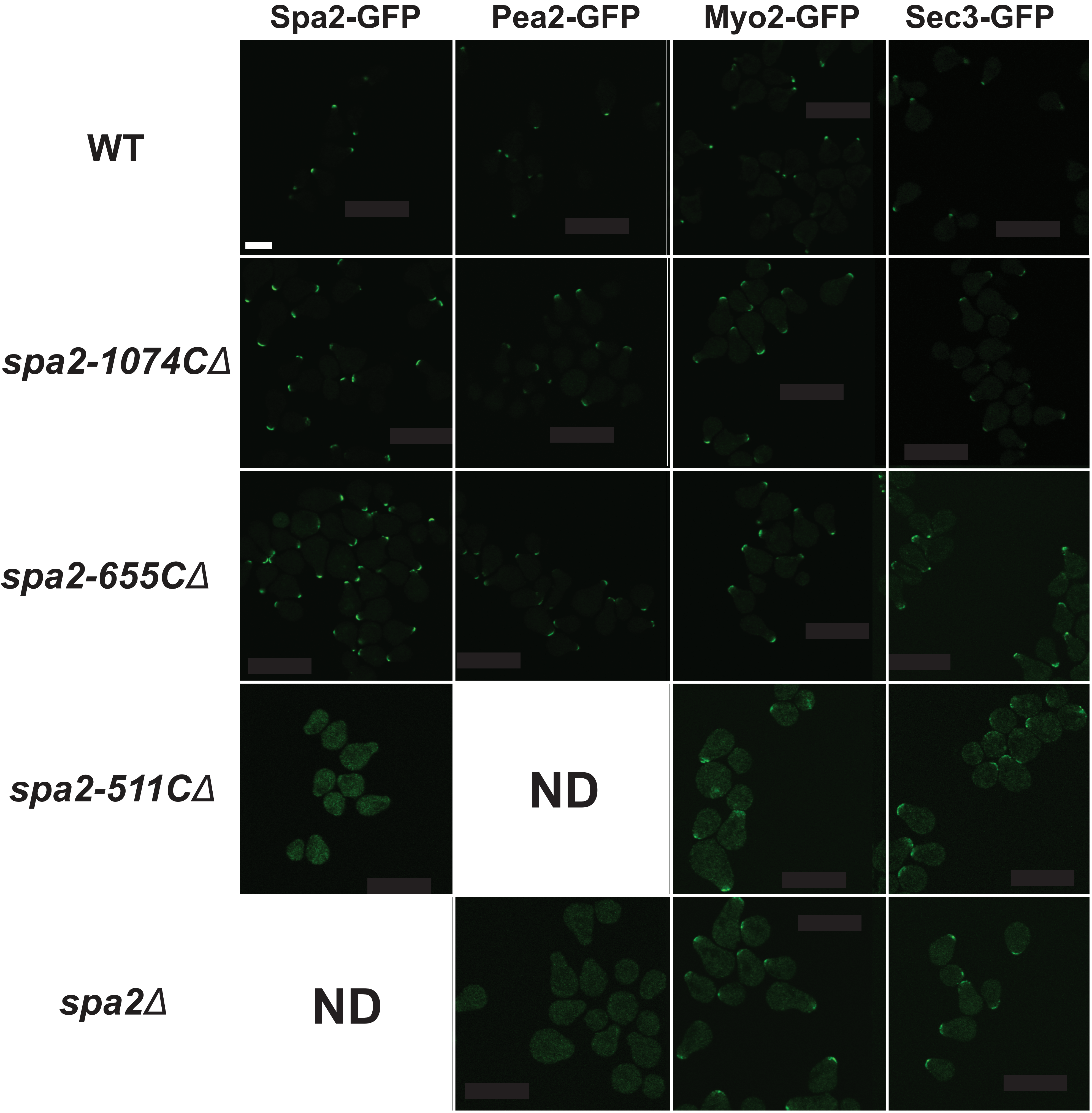


**S2 Fig.** Images of polarisome marker proteins in *spa2* deletion mutant backgrounds. In WT, *spa2-1074CΔ*, *spa2-655CΔ*, *spa2-511CΔ*, and *spa2Δ* backgrounds, the C-terminus of Spa2, Pea2, Myo2, and Sec3 were labeled with GFP. Cells were treated with α-factor for 2h, and imaged by confocal microscopy. The *spa2-511CΔ* and *spa2Δ* strains did not produce detectable Spa2-GFP or Pea2-GFP. ND indicates not determined. Scale bar = 5 μm.
