# Supplementary material for: A positive feedback loop involving the Spa2 SHD domain contributes to focal polarization
Source: PLoS One. 2022 Feb 8;17(2):e0263347. doi: 10.1371/journal.pone.0263347 (PMC8824340; doi:10.1371/journal.pone.0263347)
Supplement: S3 Fig — The width of Bni1-GFP polarization (below) was measured in microns and compared to wild-type (***, p < 0.001; **, p < 0.01; n = 20 cells). Scale bar = 5 μm. (PDF) [file pone.0263347.s003.pdf]

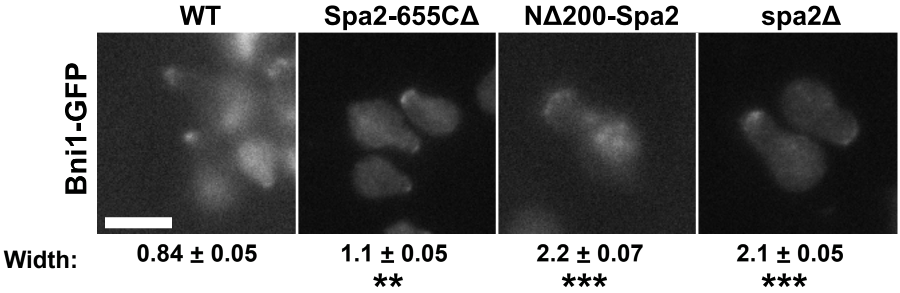

S3 Fig. Fluorescent images of wild-type and *spa2* mutant cells containing Bni1-GFP treated with α-factor for 2h. The width of Bni1-GFP polarization (below) was measured in microns and compared to wild-type (***, $p < 0.001$; **, $p < 0.01$; $n = 20$ cells). Scale bar = 5 μm.
