# Supplementary material for: A positive feedback loop involving the Spa2 SHD domain contributes to focal polarization
Source: PLoS One. 2022 Feb 8;17(2):e0263347. doi: 10.1371/journal.pone.0263347 (PMC8824340; doi:10.1371/journal.pone.0263347)
Supplement: S4 Fig — Gyp2, Msb3, Msb4, and Gyp5 were tagged with GFP in wild-type (WT), SDR14A, SDR24A, SDR124A, and spa2Δ strains. Imaging was performed after 2h pheromone treatment. Some of these data (Msb3-GFP and Msb4-GFP) are reproduced in Fig 3B of the main text. Gyp2-GFP and Gyp5-GFP show some polarization in the SDR24A strain, whereas Msb3-GFP and Msb4-GFP do not. Gyp5-GFP also shows some polarization in the SDR124A and spa2Δ strains, whereas Msb3-GFP and Msb4-GFP do not. Scale bar = 5 μm. (PDF) [file pone.0263347.s004.pdf]

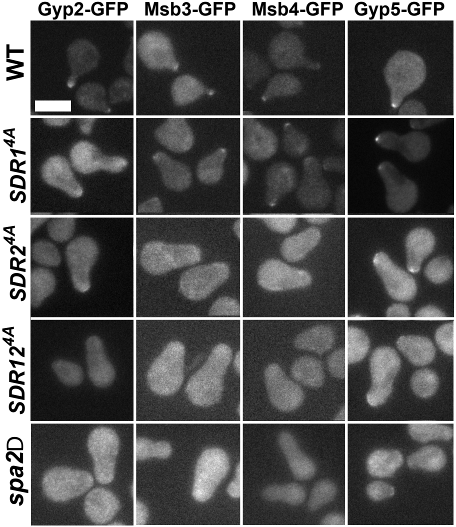

S4 Fig. Localization of Msb/Gyp proteins in *spa2* mutant backgrounds.
Gyp2, Msb3, Msb4, and Gyp5 were tagged with GFP in wild-type (WT), *SDR1*^{4A}, *SDR2*^{4A}, *SDR12*^{4A}, and *spa2Δ* strains. Imaging was performed after 2h pheromone treatment. Some of these data (Msb3-GFP and Msb4-GFP) are reproduced in Figure 3B of the main text. Gyp2-GFP and Gyp5-GFP show some polarization in the *SDR2*^{4A} strain, whereas Msb3-GFP and Msb4-GFP do not. Gyp5-GFP also shows some polarization in the *SDR12*^{4A} and *spa2Δ* strains, whereas Msb3-GFP and Msb4-GFP do not. Scale bar = 5 μm.
