# Supplementary material for: A positive feedback loop involving the Spa2 SHD domain contributes to focal polarization
Source: PLoS One. 2022 Feb 8;17(2):e0263347. doi: 10.1371/journal.pone.0263347 (PMC8824340; doi:10.1371/journal.pone.0263347)
Supplement: S5 Fig — Cells were treated with α-factor for 2 hours, and then cell extracts were prepared. Western blots were performed with anti-GFP and anti-α-tubulin antibodies, and band fluorescence was imaged using the LI-COR Odyssey system. The bar graph underneath shows quantitization of Bud6-GFP band relative to the α-tubulin band. The mutant GFP/Tubulin ratios were normalized to the wild-type ratio. The mean and standard deviation from three trials are shown. None of the mutant ratios were significantly different from wild-type by t-test. (PDF) [file pone.0263347.s005.pdf]

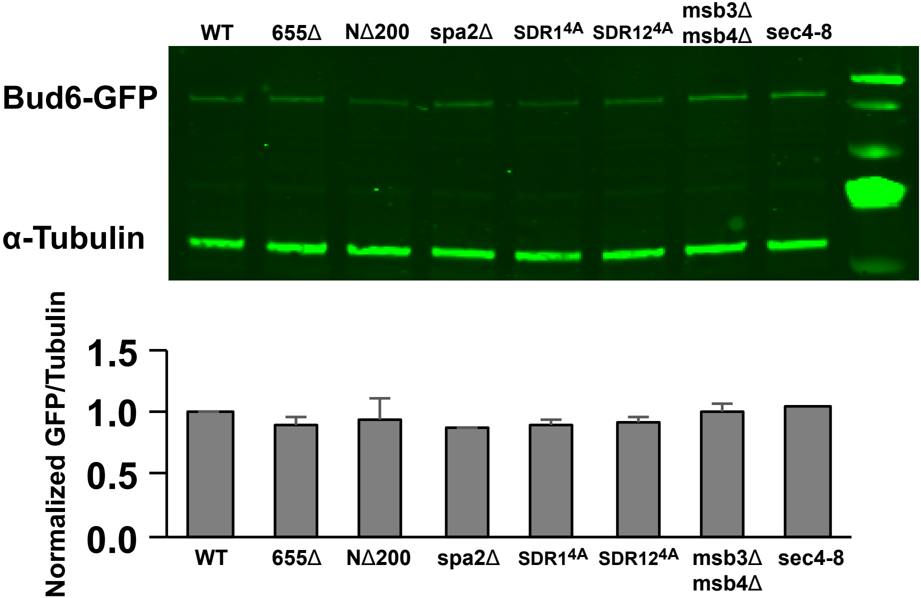

S5 Fig. Western blot of Bud6-GFP in *spa2*, *msb3/4*, and *sec4* mutant strains. Cells were treated with α-factor for 2 hours, and then cell extracts were prepared. Western blots were performed with anti-GFP and anti-α-tubulin antibodies, and band fluorescence was imaged using the LI-COR Odyssey system. The bar graph underneath shows quantitization of Bud6-GFP band relative to the α-tubulin band. The mutant GFP/Tubulin ratios were normalized to the wild-type ratio. The mean and standard deviation from three trials are shown. None of the mutant ratios were significantly different from wild-type by t-test.
