# Supplementary material for: A positive feedback loop involving the Spa2 SHD domain contributes to focal polarization
Source: PLoS One. 2022 Feb 8;17(2):e0263347. doi: 10.1371/journal.pone.0263347 (PMC8824340; doi:10.1371/journal.pone.0263347)
Supplement: S6 Fig — Cells were treated with α-factor for 2 hours, and then stained with rhodamine-phalloidin. Scale bar = 5 μm. (PDF) [file pone.0263347.s006.pdf]

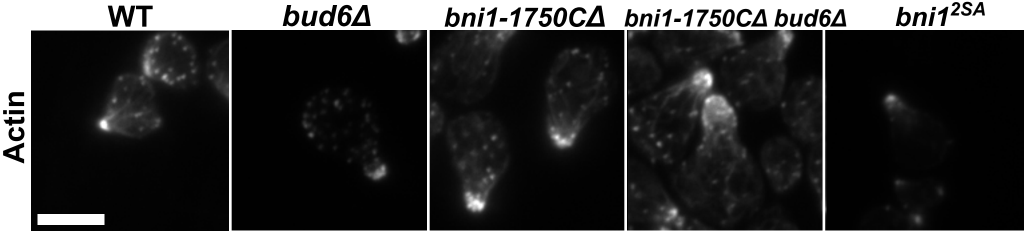

S6 Fig. Actin cytoskeleton in *bud6* and *bni1* mutant strains. Cells were treated with α-factor for 2 hours, and then stained with rhodamine-phalloidin. Scale bar = 5 μm.
