# Supplementary material for: A positive feedback loop involving the Spa2 SHD domain contributes to focal polarization
Source: PLoS One. 2022 Feb 8;17(2):e0263347. doi: 10.1371/journal.pone.0263347 (PMC8824340; doi:10.1371/journal.pone.0263347)
Supplement: S7 Fig — Msb3-GFP and GFP-Sec4 were imaged in NΔ200-Spa2-Bud6C cells after treatment with α-factor for 2h (third column) and then imaged. Scale bar = 5 μm. For comparison Msb3-GFP and GFP-Sec4 polarization in wild-type (WT, column 1) and spa2 (column 2) strains are also shown. Although the polarisome is punctate in the gain-of-function NΔ200-Spa2-Bud6C cells, the upstream components Msb3-GFP and GFP-Sec4 exhibit the more dispersed appearance found in spa2 cells. (PDF) [file pone.0263347.s007.pdf]

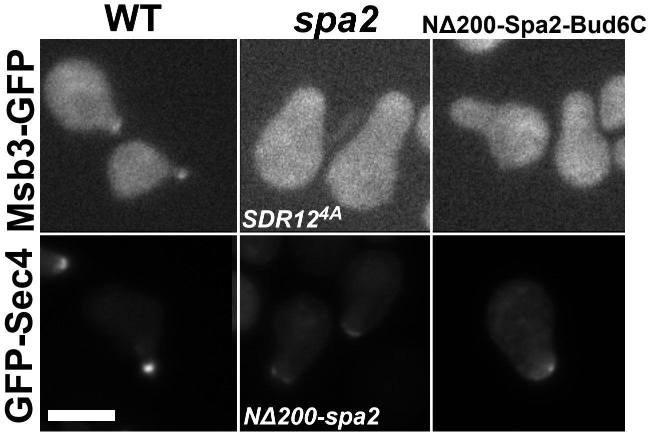

S7 Fig. Msb3-GFP and GFP-Sec4 in NΔ200-Spa2-Bud6C cells. Msb3-GFP and GFP-Sec4 were imaged in NΔ200-Spa2-Bud6C cells after treatment with α-factor for 2h (third column) and then imaged. Scale bar = 5 μm. For comparison Msb3-GFP and GFP-Sec4 polarization in wild-type (WT, column 1) and *spa2* (column 2) strains are also shown. Although the polarisome is punctate in the gain-of-function NΔ200-Spa2-Bud6C cells, the upstream components Msb3-GFP and GFP-Sec4 exhibit the more dispersed appearance found in *spa2* cells.
