# Supplementary material for: A positive feedback loop involving the Spa2 SHD domain contributes to focal polarization
Source: PLoS One. 2022 Feb 8;17(2):e0263347. doi: 10.1371/journal.pone.0263347 (PMC8824340; doi:10.1371/journal.pone.0263347)
Supplement: S8 Fig — (A) Wild-type Spa2-GFP cells were treated with 1 μM α-factor for 90 min, and then exposed to 50 μM latrunculin A for 15 min. Typical cells before (t = 0) and after LatA (t = 15m) addition are shown (fluorescence and merged fluorescence (green) and bright-field). Note that it is not the same cells in the field of view, but rather representative cells from a time-course experiment. (B) Co-localization of Spa2-mCherry with Myo2-GFP, Pea2-GFP, and GFP-Sec4 after LatA exposure and washout. Cells were treated with α-factor for 1h, and then exposed to LatA for 15m (+LatA), followed by removal of LatA for 15m (-LatA) and continued α-factor treatment throughout. Representative cells are shown in this time-course experiment from the GFP and mCherry channels as well as the merged images. (C) Time-lapse experiment in which Myo2-GFP/Spa2-mCherry cells were continually treated with 1 μM α-factor. After 1h initial treatment, LatA was introduced into the culture chamber for 15m (+LatA), and then washed out with α-factor containing YPD, followed by imaging for another 15m (-LatA). The same cells were followed in the experiment with the cell periphery outlined in the dashed white lines. GFP, mCherry, and merged images are shown. In all the experiments, Spa2 polarizes to the polarisome during initial mating factor response, de-polarizes during LatA treatment, and then re-polarizes to polarisome after LatA is removed. Myo2, Pea2, and Sec4 co-localize with Spa2 during these dynamics. Scale bar = 5 μm. (PDF) [file pone.0263347.s008.pdf]

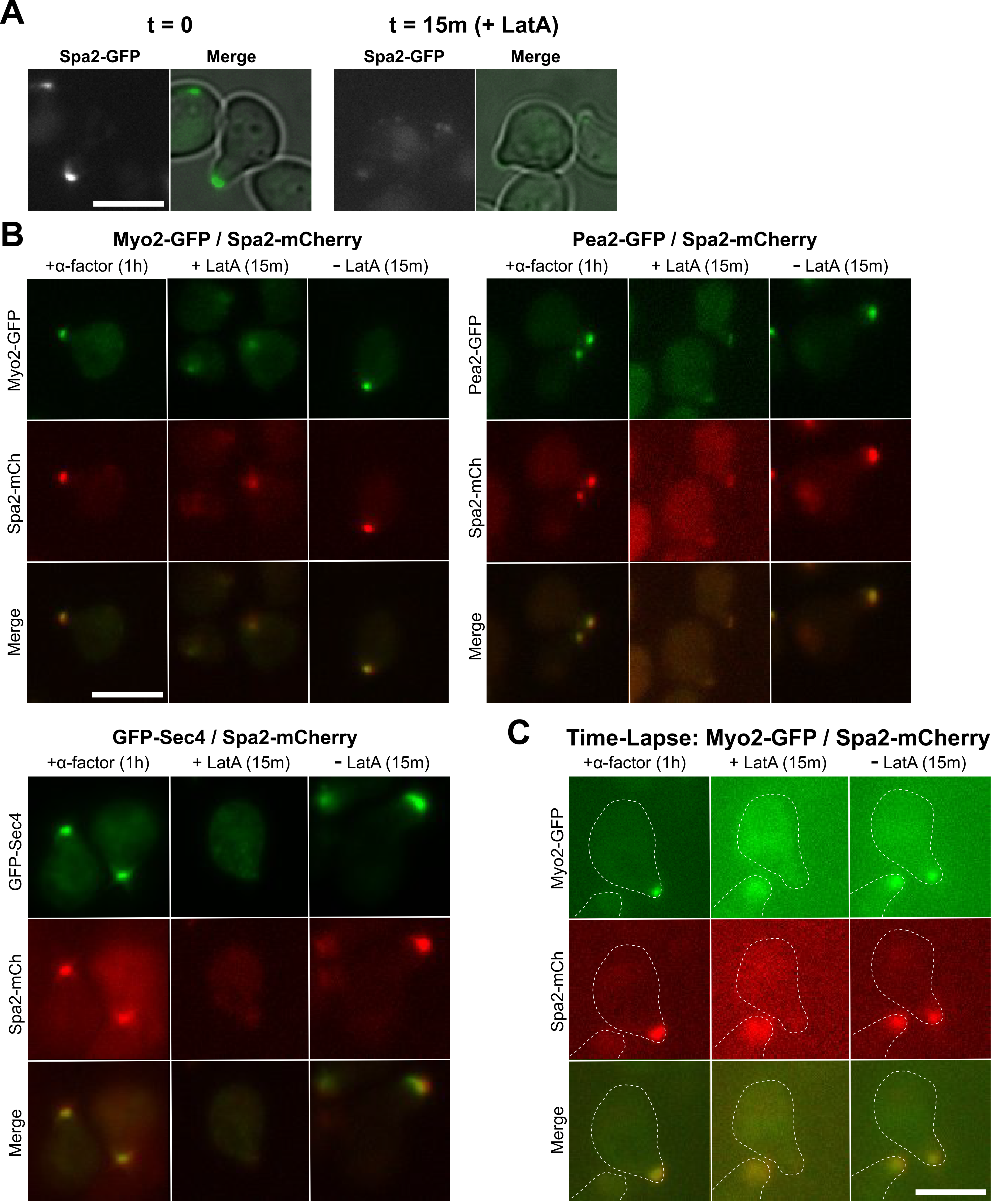

S8 Fig. Effect of latrunculin A (LatA) on Spa2 polarization. **(A)** Wild-type Spa2-GFP cells were treated with 1 μM α-factor for 90 min, and then exposed to 50 μM latrunculin A for 15 min. Typical cells before (t = 0) and after LatA (t = 15m) addition are shown (fluorescence and merged fluorescence (green) and bright-field). Note that it is not the same cells in the field of view, but rather representative cells from a time-course experiment. **(B)** Co-localization of Spa2-mCherry with Myo2-GFP, Pea2-GFP, and GFP-Sec4 after LatA exposure and washout. Cells were treated with α-factor for 1h, and then exposed to LatA for 15m (+ LatA), followed by removal of LatA for 15m (- LatA) and continued α-factor treatment throughout. Representative cells are shown in this time-course experiment from the GFP and mCherry channels as well as the merged images. **(C)** Time-lapse experiment in which Myo2-GFP/Spa2-mCherry cells were continually treated with 1 μM α-factor. After 1h initial treatment, LatA was introduced into the culture chamber for 15m (+ LatA), and then washed out with α-factor containing YPD, followed by imaging for another 15m (- LatA). The same cells were followed in the experiment with the cell periphery outlined in the dashed white lines. GFP, mCherry, and merged images are shown. In all the experiments, Spa2 polarizes to the polarisome during initial mating factor response, de-polarizes during LatA treatment, and then re-polarizes to polarisome after LatA is removed. Myo2, Pea2, and Sec4 co-localize with Spa2 during these dynamics. Scale bar = 5 μm.
