# Supplementary material for: A positive feedback loop involving the Spa2 SHD domain contributes to focal polarization
Source: PLoS One. 2022 Feb 8;17(2):e0263347. doi: 10.1371/journal.pone.0263347 (PMC8824340; doi:10.1371/journal.pone.0263347)
Supplement: S10 Fig — Left: time-series visualization of spatial localization of Spa2 on the membrane (in degrees) on the y-axis and time on the x-axis. Molecule numbers are color-coded by the color bar. Right upper: Numbers of Spa2 on the membrane as a function of time for a sample simulation. Right lower: snapshot of spatial profile of Spa2 on the membrane at a sample time point after polarization. (PDF) [file pone.0263347.s010.pdf]

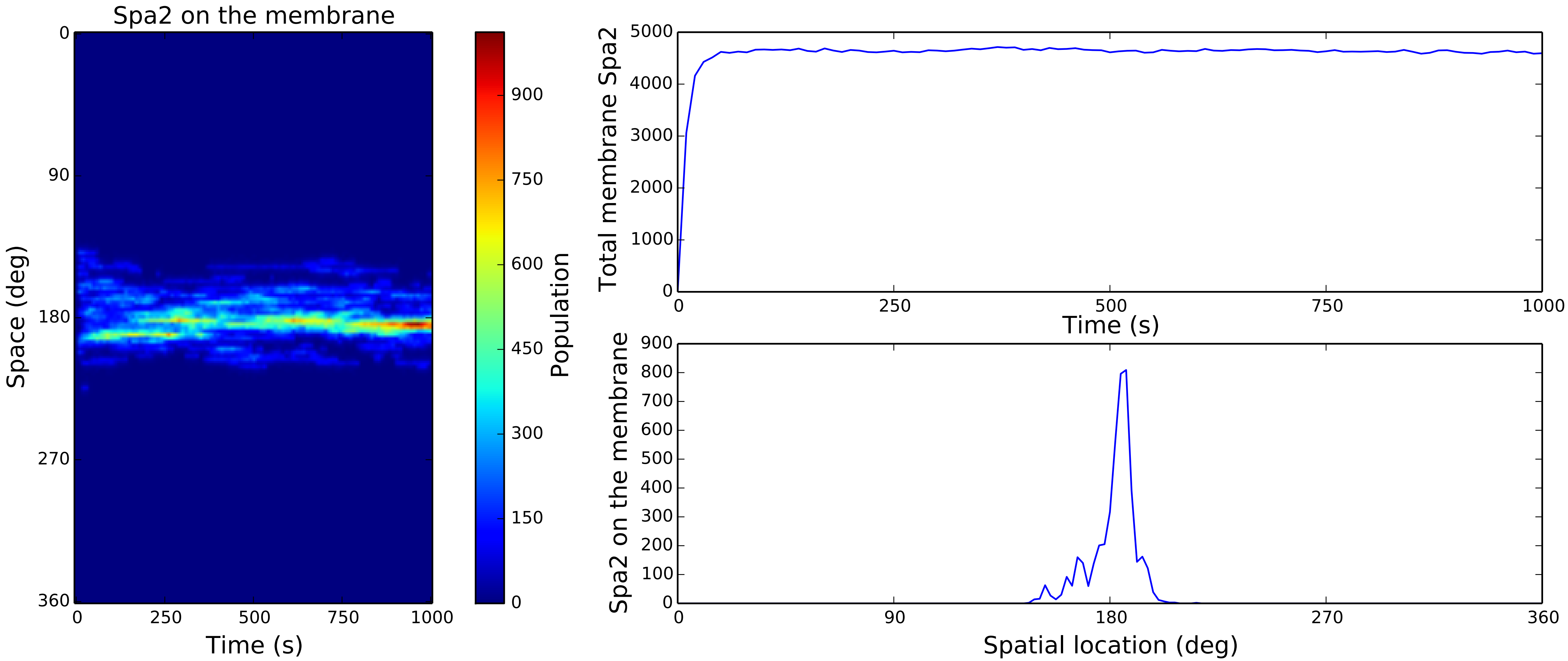

S10 Fig. Spa2 spatial dynamics in Bud6 model simulations. **Left:** time-series visualization of spatial localization of Spa2 on the membrane (in degrees) on the y-axis and time on the x-axis. Molecule numbers are color-coded by the color bar. **Right upper:** Numbers of Spa2 on the membrane as a function of time for a sample simulation. **Right lower:** snapshot of spatial profile of Spa2 on the membrane at a sample time point after polarization.
