# Supplementary material for: A positive feedback loop involving the Spa2 SHD domain contributes to focal polarization
Source: PLoS One. 2022 Feb 8;17(2):e0263347. doi: 10.1371/journal.pone.0263347 (PMC8824340; doi:10.1371/journal.pone.0263347)
Supplement: S11 Fig — Parameter sweep has the Km values on the x-axis and the ratio of Bfb/Bon on the y-axis. (A) Regions of parameter space with tight polarization and accurate tracking. Red indicates areas where the width is sufficiently polarized (FWHM < 18 degrees); blue indicates areas where tracking probability is greater than 70%; Purple indicates areas where both conditions are satisfied. (B) Width of the polarisome measured as the FWHM as a best-fit Gaussian. (C) Absolute uncertainty in the width measurement. (D) Probability of successfully tracking a moving input. (E) Absolute uncertainty in the tracking probability. (PDF) [file pone.0263347.s011.pdf]

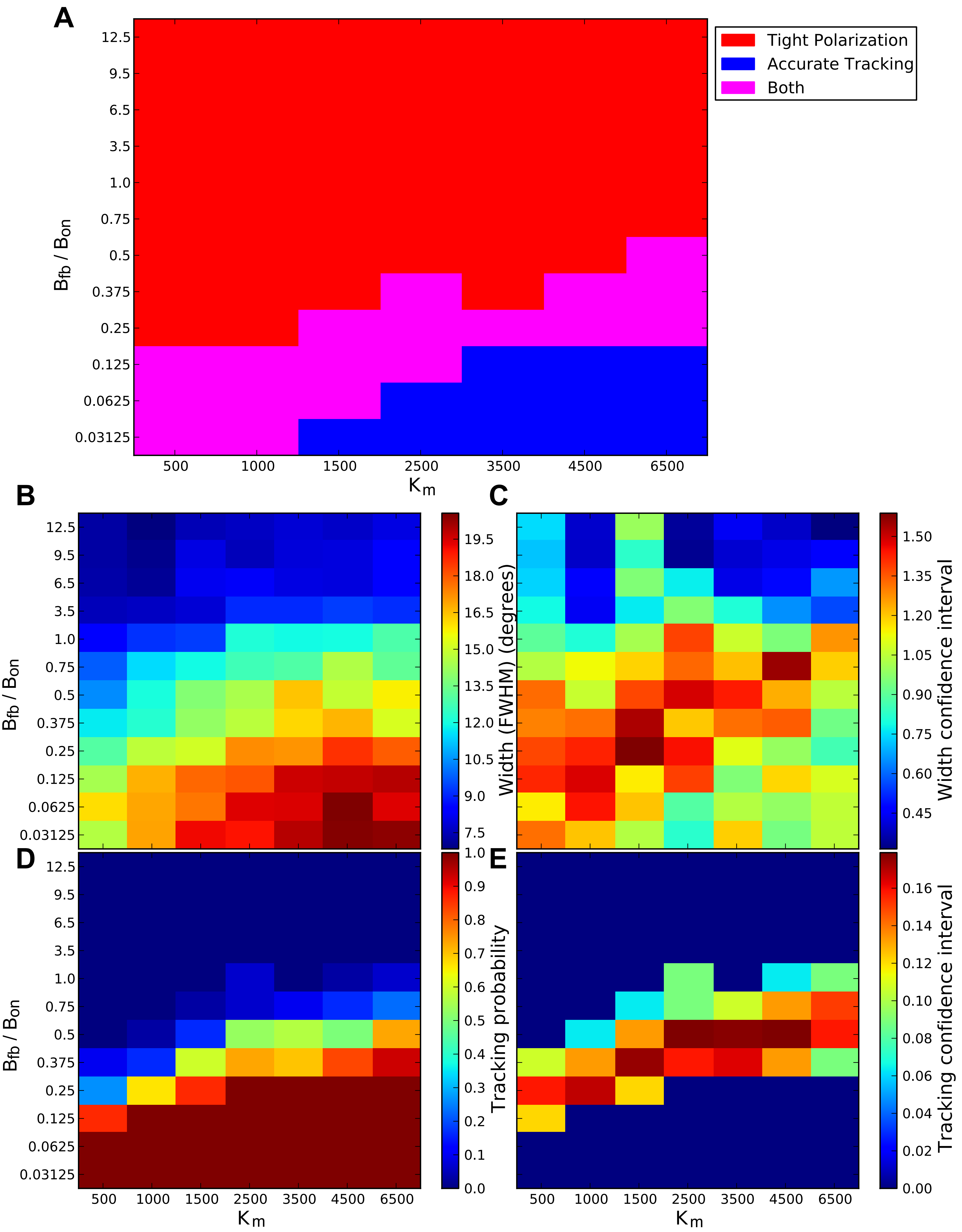

S11 Fig. Results from a parameter sweep of the default polarisome model. Parameter sweep has the K_m values on the x-axis and the ratio of B_{fb}/B_{on} on the y-axis. **(A)** Regions of parameter space with tight polarization and accurate tracking. Red indicates areas where the width is sufficiently polarized (FWHM ≤ 18 degrees); blue indicates areas where tracking probability is greater than 70%; Purple indicates areas where both conditions are satisfied. **(B)** Width of the polarisome measured as the FWHM as a best-fit Gaussian. **(C)** Absolute uncertainty in the width measurement. **(D)** Probability of successfully tracking a moving input. **(E)** Absolute uncertainty in the tracking probability.
