# Supplementary figures and images for: A positive feedback loop involving the Spa2 SHD domain contributes to focal polarization
Source: PLoS One. 2022 Feb 8;17(2):e0263347. doi: 10.1371/journal.pone.0263347 (PMC8824340; doi:10.1371/journal.pone.0263347)

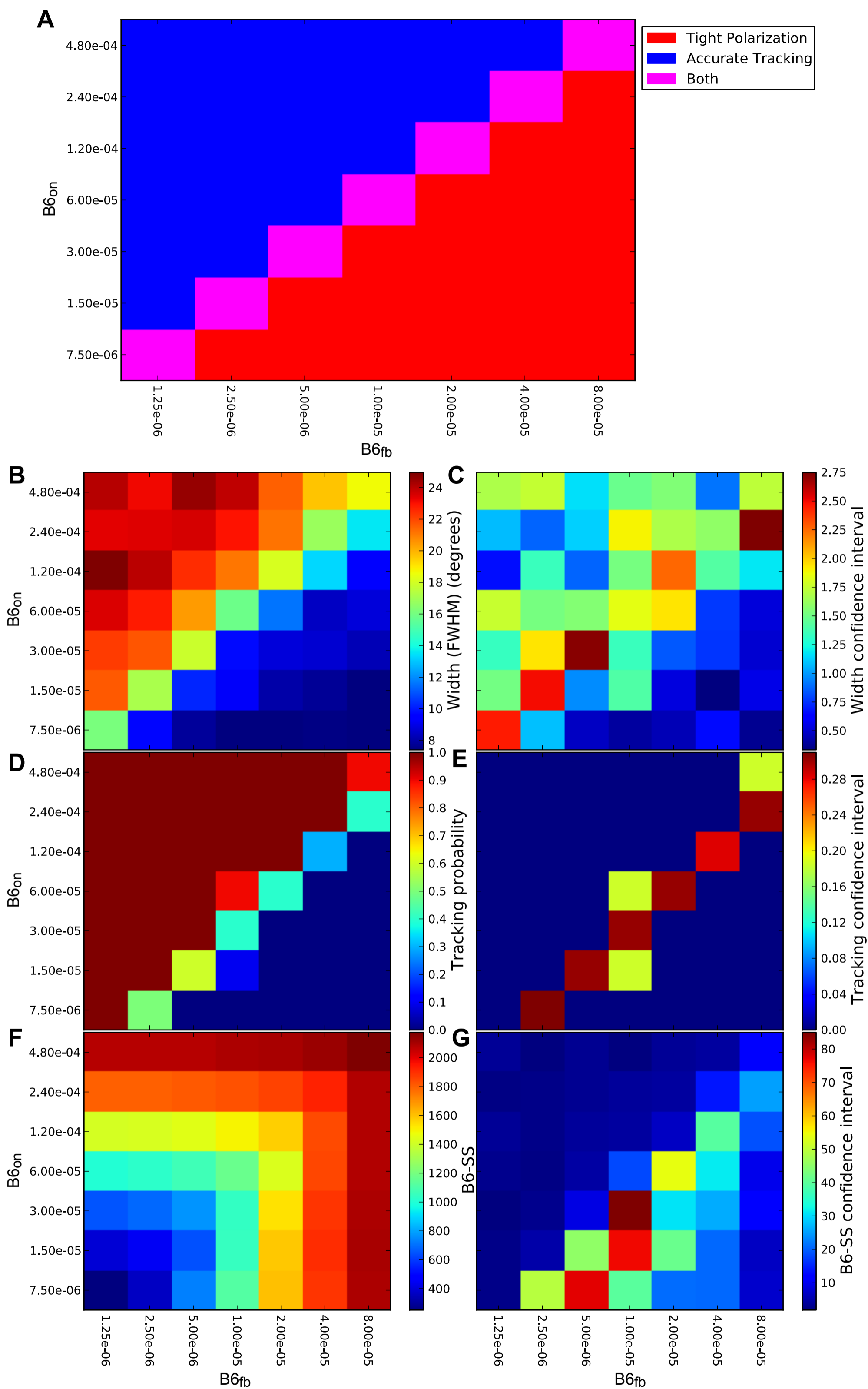

Supplement: S12 Fig — Parameter sweep has the B6fb parameter values on the x-axis and the B6on parameter values on the y-axis. (A) Regions of parameter space with tight polarization and accurate tracking. Red indicates areas where the width is sufficiently polarized (FWHM < 18 degrees); blue indicates areas where tracking probability is greater than 70%; Purple indicates areas where both conditions are satisfied. (B) Width of the polarisome measured as the FWHM as a best-fit Gaussian. (C) Absolute uncertainty in the width measurement. (D) Probability of successfully tracking a moving input. (E) Absolute uncertainty in the tracking probability. (F) Number of Bud6 molecules on the membrane at steady-state (Bud6-SS). (G) Absolute uncertainty in Bud6-SS numbers. (PDF) [file pone.0263347.s012.pdf]
