# Supplementary material for: A positive feedback loop involving the Spa2 SHD domain contributes to focal polarization
Source: PLoS One. 2022 Feb 8;17(2):e0263347. doi: 10.1371/journal.pone.0263347 (PMC8824340; doi:10.1371/journal.pone.0263347)
Supplement: S1 Table — (PDF) [file pone.0263347.s013.pdf]

S1 Table. Alpha-factor halo assay on wild-type and mutant strains.

Strain	0.01 µg	0.03 µg	0.1 µg
Wild-Type	8.2 ± 1.3	12.5 ± 0.5	16.8 ± 0.8
spa2Δ	6.8 ± 0.3	11.8 ± 0.8	16.2 ± 0.8
NΔ200-spa2	8.0 ± 0.5	13.3 ± 1.4	17.5 ± 1.0
SDR12^{4A}	7.7 ± 1.8	13.3 ± 1.4	17.2 ± 2.0
msb3Δ msb4Δ	7.5 ± 0.5	12.8 ± 0.3	16.5 ± 0.9

Columns are amount of alpha-factor spotted on disc. Mean halo size and standard deviation (mm) for three trials. None of the mutant values were significantly different from wild-type by t-test.
