# Supplementary material for: A positive feedback loop involving the Spa2 SHD domain contributes to focal polarization
Source: PLoS One. 2022 Feb 8;17(2):e0263347. doi: 10.1371/journal.pone.0263347 (PMC8824340; doi:10.1371/journal.pone.0263347)
Supplement: S2 Table — (PDF) [file pone.0263347.s014.pdf]

S2 Table. Biochemical species and initial conditions for the polarisome models.

**Species**	**Description**	**Initial Conditions**
$Bni1_c$	Bni1 in the cytoplasm	1000
$Bni1_m$	Bni1 on the plasma membrane	0
$Spa2_c$	Spa2 in the cytoplasm	5000
$Spa2_m$	Spa2 on the plasma membrane	0
$Bud6_c$	Bud6 in the cytoplasm	2500
$Bud6_m$	Bud6 on the plasma membrane	0
$Actin_c$	Actin in the cytoplasm	40
$Actin_m$	Actin on the plasma membrane	0
