# Supplementary material for: A positive feedback loop involving the Spa2 SHD domain contributes to focal polarization
Source: PLoS One. 2022 Feb 8;17(2):e0263347. doi: 10.1371/journal.pone.0263347 (PMC8824340; doi:10.1371/journal.pone.0263347)
Supplement: S3 Table — (PDF) [file pone.0263347.s015.pdf]

S3 Table. Reactions and reaction rates for the original and Bud6 polarisome models.

Reaction	Reaction Rate	Description/References
$Bni1_c + Cdc42_m \rightarrow Bni1_m + Cdc42_m$	$B_{on} \times [Bni1_c] \times [Cdc42_m]$	Active Cdc42 recruits Bni1 to the membrane [2]
$Bni1_m \rightarrow Bni1_c$	$B_{off} \times [Bni1_m]$	Bni1 dissociates from the membrane via actin patches [1]
$Actin_c + Bni1_m \rightarrow Actin_m + Bni1_m$	$A_{on} \times [Bni1_m] \times [Bud6_m] \times [Actin_c]$	Bni1 initiates polymerization of actin cables on the membrane [3]
$Actin_m + Spa2_m \rightarrow Actin_c + Spa2_m$	$A_{off} \frac{K_m}{K_m + [Spa2_m]} \times [Actin_m]$	Spa2 inhibits actin depolymerization
$Spa2_c + Actin_m \rightarrow Spa2_m + Actin_m$	$S_{on} \times [Spa2_c] \times [Actin_m]$	Spa2 is transported along actin cables to the membrane [5]
$Spa2_m \rightarrow Spa2_c$	$S_{off} \times [Spa2_m]$	Spa2 dissociates from the membrane
$Bni1_c + Spa2_m \rightarrow Bni1_m + Spa2_m$	$B_{fb} \times [Bni1_c] \times [Spa2_m]$	Spa2 on the membrane recruits Bni1 to the membrane [5]
$Bud6_c + Cdc42_m \rightarrow Bud6_m + Cdc42_m$	$B6_{on} \times [Bud6_c] \times [Cdc42_m]$	Active Cdc42 recruits Bud6 to the membrane
$Bud6_c + Spa2_m \rightarrow Bud6_m + Spa2_m$	$B6_{fb} \times [Bud6_c] \times [Spa2_m] \times [Actin_m]$	Spa2 recruits Bud6 to the membrane
$Bud6_m \rightarrow Bud6_c$	$B6_{off} \times [Bud6_m]$	Bud6 dissociates from the membrane

The reaction rates are all first-, second-, or third-order kinetics except for the actin depolymerization reaction which contains an inhibition term. The Bud6-specific reactions are below the double line.
