# Supplementary material for: A positive feedback loop involving the Spa2 SHD domain contributes to focal polarization
Source: PLoS One. 2022 Feb 8;17(2):e0263347. doi: 10.1371/journal.pone.0263347 (PMC8824340; doi:10.1371/journal.pone.0263347)
Supplement: S4 Table — (PDF) [file pone.0263347.s016.pdf]

S4 Table. Parameters and parameter values for the original and Bud6 polarisome models.

Parameter	Value
B_{on}	1.6 x 10^{-6} $mol^{-1}s^{-1}$
B_{off}	0.25 s^{-1}
B_{fb}	1.9 x 10^{-5} $mol^{-1}s^{-1}$
A_{on}	7.7 x 10^{-5} $mol^{-1}s^{-1}$
A_{off}	0.018 s^{-1}
K_m	3500 mol
S_{on}	0.16 $mol^{-1}s^{-1}$
S_{off}	0.35 s^{-1}
$B6_{on}$	6 x 10^{-5} $mol^{-1}s^{-1}$
$B6_{fb}$	1 x 10^{-5} $mol^{-1}s^{-1}$
$B6_{off}$	0.5 $mol^{-1}s^{-1}$
$Bni1_t$	1000 molecules
$Spa2_t$	5000 molecules
$Bud6_t$	2500 molecules
$Actin_t$	40 cables (molecules)
D	0.0053 $\mu m^2/s$
