# Supplementary material for: A positive feedback loop involving the Spa2 SHD domain contributes to focal polarization
Source: PLoS One. 2022 Feb 8;17(2):e0263347. doi: 10.1371/journal.pone.0263347 (PMC8824340; doi:10.1371/journal.pone.0263347)
Supplement: S5 Table — (PDF) [file pone.0263347.s017.pdf]

S5 Table. Yeast strains used in this study

Strain	Genotype	Source
RJD415	*MAT***a** *can1-100 leu2-3-112 his3-11-15 trp1-1 ura3-1 ade2-1 pep4Δ::TRP1 bar1Δ::LEU2*	Ray Deshaies
TMY431	RJD415 *spa2Δ::KAN*^{R}	
TMY900	RJD415 *spa2Δ::SPA2-GFP-HIS5*	This study
TMY901	RJD415 *spa2Δ::SPA2-1074CΔ-GFP-HIS5*	This study
TMY902	RJD415 *spa2Δ::SPA2-655CΔ-GFP-HIS5*	This study
TMY903	RJD415 *spa2Δ::SPA2-511CΔ-GFP-HIS5*	This study
TMY904	RJD415 *spa2Δ::NΔ200-SPA2-KAN*^{R}	This study
TMY905	RJD415 *spa2Δ::NΔ200-SPA2-GFP-HIS5*	This study
TMY906	RJD415 *spa2Δ::NΔ400-SPA2-GFP-HIS5*	This study
TMY907	RJD415 *spa2Δ::SPA2-1074CΔ-KAN*^{R} *pea2Δ::PEA2-GFP-HIS5*	This study
TMY908	RJD415 *spa2Δ::SPA2-655CΔ-KAN*^{R} *pea2Δ::PEA2-GFP-HIS5*	This study
TMY909	RJD415 *spa2Δ::SPA2-511CΔ-KAN*^{R} *pea2Δ::PEA2-GFP-HIS5*	This study
TMY910	RJD415 *spa2Δ::NΔ200-SPA2-KAN*^{R} *pea2Δ::PEA2-GFP-HIS5*	This study
TMY911	RJD415 *sec3Δ::SEC3-GFP-HIS5*	This study
TMY912	RJD415 *spa2Δ::SPA2-1074CΔ-KAN*^{R} *sec3Δ::SEC3-GFP-HIS5*	This study
TMY913	RJD415 *spa2Δ::SPA2-655CΔ-KAN*^{R} *sec3Δ::SEC3-GFP-HIS5*	This study
TMY914	RJD415 *spa2Δ::SPA2-511CΔ-KAN*^{R} *sec3Δ::SEC3-GFP-HIS5*	This study
TMY915	RJD415 *spa2Δ::NΔ200-SPA2-KAN*^{R} *sec3Δ::SEC3-GFP-HIS5*	This study
TMY916	RJD415 *spa2Δ::NΔ400-SPA2-KAN*^{R} *sec3Δ::SEC3-GFP-HIS5*	This study
TMY917	RJD415 *spa2*$^{SDR1\_4A}$	This study
TMY918	RJD415 *spa2*$^{SDR2\_4A}$	This study
TMY919	RJD415 *spa2*$^{SDR12\_4A}$	This study
TMY920	RJD415 *spa2*$^{SDR1\_4A}$*:: GFP-HIS5*	This study
TMY921	RJD415 *spa2*$^{SDR2\_4A}$*::GFP-HIS5*	This study
TMY922	RJD415 *spa2*$^{SDR12\_4A}$*::GFP-HIS5*	This study
TMY923	RJD415 *fus1Δ::FUS1-GFP-HIS5*	This study
TMY924	RJD415 *fus1Δ::FUS1-GFP-HIS5 spa2Δ::SPA2-655CΔ-HIS5*	This study
TMY925	RJD415 *fus1Δ::FUS1-GFP-HIS5 spa2Δ:: NΔ200-SPA2-KAN*^{R}	This study
TMY926	RJD415 *fus1Δ::FUS1-GFP-HIS5 spa2Δ:: KAN*^{R}	This study
TMY927	RJD415 *fus1Δ::FUS1-GFP-HIS5 spa2Δ::SPA2-mCHERRY-KAN*^{R}	This study
TMY928	RJD415 *gyp1Δ::GYP1-GFP-HIS5 spa2Δ::SPA2-mCHERRY-KAN*^{R}	This study
TMY929	RJD415 *gyp2Δ::GYP2-GFP-HIS5 spa2Δ::SPA2-mCHERRY-KAN*^{R}	This study
TMY930	RJD415 *msb3Δ::MSB3-GFP-HIS5 spa2Δ::SPA2-mCHERRY-KAN*^{R}	This study
TMY931	RJD415 *msb4Δ::MSB4-GFP-HIS5 spa2Δ::SPA2-mCHERRY-KAN*^{R}	This study
TMY932	RJD415 *gyp5Δ::GYP5-GFP-HIS5 spa2Δ::SPA2-mCHERRY-KAN*^{R}	This study
TMY933	RJD415 *gyp6Δ::GYP6-GFP-HIS5 spa2Δ::SPA2-mCHERRY-KAN*^{R}	This study
TMY934	RJD415 *gyp7Δ::GYP7-GFP-HIS5 spa2Δ::SPA2-mCHERRY-KAN*^{R}	This study
TMY935	RJD415 *gyp8Δ::GYP8-GFP-HIS5 spa2Δ::SPA2-mCHERRY-KAN*^{R}	This study
TMY936	RJD415 *gyp1Δ:: KAN*^{R}	This study

TMY937	RJD415 *gyp2Δ:: KAN*^{R}	This study
TMY938	RJD415 *msb3Δ:: KAN*^{R}	This study
TMY939	RJD415 *msb4Δ:: KAN*^{R}	This study
TMY940	RJD415 *gyp5Δ:: KAN*^{R}	This study
TMY941	RJD415 *gyp6Δ:: KAN*^{R}	This study
TMY942	RJD415 *gyp7Δ:: KAN*^{R}	This study
TMY943	RJD415 *gyp8Δ:: KAN*^{R}	This study
TMY944	RJD415 *spa2*^{SDR14A} *gyp2Δ::GYP2-GFP-HIS5*	This study
TMY945	RJD415 *spa2*^{SDR14A} *msb3Δ::MSB3-GFP-HIS5*	This study
TMY946	RJD415 *spa2*^{SDR14A} *msb4Δ::MSB4-GFP-HIS5*	This study
TMY947	RJD415 *spa2*^{SDR14A} *gyp5Δ::GYP5-GFP-HIS5*	This study
TMY948	RJD415 *spa2*^{SDR24A} *gyp2Δ::GYP2-GFP-HIS5*	This study
TMY949	RJD415 *spa2*^{SDR24A} *msb3Δ::MSB3-GFP-HIS5*	This study
TMY950	RJD415 *spa2*^{SDR24A} *msb4Δ::MSB4-GFP-HIS5*	This study
TMY951	RJD415 *spa2*^{SDR24A} *gyp5Δ::GYP5-GFP-HIS5*	This study
TMY952	RJD415 *spa2*^{SDR124A} *gyp2Δ::GYP2-GFP-HIS5*	This study
TMY953	RJD415 *spa2*^{SDR124A} *msb3Δ::MSB3-GFP-HIS5*	This study
TMY954	RJD415 *spa2*^{SDR124A} *msb4Δ::MSB4-GFP-HIS5*	This study
TMY955	RJD415 *spa2*^{SDR124A} *gyp5Δ::GYP5-GFP-HIS5*	This study
TMY956	RJD415 *msb3Δ:: KAN*^{R} *msb4Δ::URA3*^{Kl}	This study
TMY957	RJD415 *msb3Δ:: KAN*^{R} *msb4Δ::URA3*^{Kl} *spa2Δ::SPA2-GFP-HIS5*	This study
TMY958	RJD415 *msb3Δ:: KAN*^{R} *msb4Δ::URA3*^{Kl} *fus1Δ::FUS1-GFP-HIS5*	This study
TMY959	RJD415 *msb3Δ:: KAN*^{R} *msb4Δ::URA3*^{Kl} *bud6Δ::BUD6-GFP-HIS5*	This study
TMY960	RJD415 *msb3Δ:: KAN*^{R} *msb4Δ::URA3*^{Kl} *[*P_{SEC4}*-GFP-SEC4 pRS316]*	This study
TMY961	RJD415 *spa2Δ::NΔ200-SPA2-KAN*^{R} *[*P_{SEC4}*-GFP-SEC4 pRS316]*	This study
TMY962	RJD415 *sec4*^{Q79L}	This study
TMY963	RJD415 *sec4*^{Q79L} *spa2Δ::SPA2-GFP-HIS5*	This study
TMY964	RJD415 *sec4*^{Q79L} *fus1Δ::FUS1-GFP-HIS5*	This study
TMY965	RJD415 *sec4Δ:: KAN*^{R} *[sec4-8 pRS316]*	This study
TMY966	RJD415 *sec4Δ:: KAN*^{R} *[sec4-8 pRS316] spa2Δ::SPA2-GFP-HIS5*	This study
TMY967	RJD415 *sec4Δ:: KAN*^{R} *[sec4-8 pRS316] fus1Δ::FUS1-GFP-HIS5*	This study
TMY968	RJD415 *sec4Δ:: KAN*^{R} *[sec4-8 pRS316] bud6Δ::BUD6-GFP-HIS5*	This study
TMY969	RJD415 *spa2Δ::SPA2-mCHERRY-KAN*^{R} *[*P_{SEC4}*-GFP-SEC4 pRS316]*	This study
TMY970	RJD415 *bud6Δ::BUD6-GFP-HIS5*	This study
TMY971	RJD415 *spa2Δ::KAN*^{R} *bud6Δ::BUD6-GFP-HIS5*	This study
TMY972	RJD415 *spa2Δ::SPA2-655CΔ-KAN*^{R} *bud6Δ::BUD6-GFP-HIS5*	This study
TMY973	RJD415 *spa2Δ::NΔ200-SPA2-KAN*^{R} *bud6Δ::BUD6-GFP-HIS5*	This study
TMY974	RJD415 *spa2*$^{SDR1\_4A}$ *bud6Δ::BUD6-GFP-HIS5*	This study
TMY975	RJD415 *spa2*$^{SDR12\_4A}$ *bud6Δ::BUD6-GFP-HIS5*	This study
TMY976	RJD415 *bud6Δ::BUD6-GFP-HIS5 spa2Δ::SPA2-mCHERRY-KAN*^{R}	This study
TMY977	RJD415 *bud6Δ::KAN*^{R} *spa2Δ::SPA2-GFP-HIS5*	This study
TMY978	RJD415 *bni1Δ:: BNI-1750CΔ- KAN*^{R}	This study
TMY979	RJD415 *bni1Δ:: BNI-1750CΔ- KAN*^{R} *spa2Δ::SPA2-GFP-HIS5*	This study

TMY980	RJD415 *bni1Δ:: BNI-1750CΔ- KAN*^{R} *bud6Δ::URA3*^{Kl}	This study
TMY981	RJD415 *bni1Δ:: BNI-1750CΔ- KAN*^{R} *bud6Δ::URA3*^{Kl} *spa2Δ::SPA2-GFP-HIS5*	This study
TMY982	RJD415 *spa2Δ::NΔ200-SPA2-GFP-KAN*^{R} *pea2Δ::PEA2-SHD-HIS5*	This study
TMY983	RJD415 *spa2Δ::NΔ200-SPA2-GFP-KAN*^{R} *ura3::P*$_{SPA2}$*-SHD-URA3*	This study
TMY984	RJD415 *spa2Δ::NΔ200-SPA2-MSB3-URA3*^{Kl} *pea2Δ::PEA2-SHD-HIS5*	This study
TMY985	RJD415 *spa2Δ::NΔ200-SPA2-KAN*^{R} *ura3::P*$_{SPA2}$*-MSB3-URA3 pea2Δ::PEA2-SHD-HIS5*	This study
TMY986	RJD415 *spa2Δ::NΔ200-SPA2-MSB3-URA3*^{K} *bud6Δ::BUD6-GFP-HIS5*	This study
TMY987	RJD415 *spa2Δ::NΔ200-SPA2-BUD6C-URA3*^{Kl} *pea2Δ::PEA2-SHD-HIS5*	This study
TMY988	RJD415 *spa2Δ::NΔ200-SPA2-KAN*^{R} *ura3::P*$_{SPA2}$*-BUD6C-URA3 pea2Δ::PEA2-SHD-HIS5*	This study
TMY989	RJD415 *spa2Δ::NΔ200-SPA2-BUD6C-URA3*^{Kl} *msb3Δ::MSB3-GFP-HIS5*	This study
TMY990	RJD415 *spa2Δ::NΔ200-SPA2-BUD6C-HIS5 [P*$_{SEC4}$*-GFP-SEC4 pRS316]*	This study
