# Supplementary material for: A positive feedback loop involving the Spa2 SHD domain contributes to focal polarization
Source: PLoS One. 2022 Feb 8;17(2):e0263347. doi: 10.1371/journal.pone.0263347 (PMC8824340; doi:10.1371/journal.pone.0263347)
Supplement: S1 Text — (PDF) [file pone.0263347.s018.pdf]

S1 Text. Original and Modified (Bud6) Model of Yeast Polarisome during Pheromone Response

We first present the polarisome model originally described in earlier work (1):

$$\frac{\partial Bni1_m}{\partial t} = \nabla^2 Bni1_m + B_{on} Bni1_c Cdc42_m + B_{fb} Bni1_c Spa2_m - B_{off} Bni1_m \qquad \text{(S1-1)}$$

$$\frac{\partial Actin_m}{\partial t} = A_{on} Bni1_m Actin_c - A_{off} \frac{K_m}{K_m + Spa2_m} Actin_m \qquad \text{(S1-2)}$$

$$\frac{\partial Spa2_m}{\partial t} = \nabla^2 Spa2_m + S_{on} Spa2_c Actin_m - S_{off} Spa2_m \qquad \text{(S1-3)}$$

The structure of the model was based on the work of Altschuler and colleagues (2)

This model was used to perform the simulations shown in Fig. 2 and S1 Fig. Two different sets of parameters were chosen, while all other parameters were set at their default values (1). In one set of simulations, B_{fb}/B_{on} and K_m were low; in the other set B_{fb}/B_{on} and K_m were chosen to be high values. Note that in the spa2Δ simulations, the total amount of Spa2 was set to ½ the wild-type number instead of 0 to reflect the presence of related proteins such as Sph1 that can potentially substitute for Spa2.

Second we present an updated version of the polarisome model in which we have added the Bud6 species and its dynamics. Bud6 acts to catalyze actin polymerization by Bni1. Eq. S2-2 has been modified from Eq. S1-1 to include Bud6 stimulation of actin cable formation. Eq. S2-4 describes the Bud6 spatial dynamics on the membrane.

$$\frac{\partial Bni1_m}{\partial t} = \nabla^2 Bni1_m + B_{on} Bni1_c Cdc42_m - B_{off} Bni1_m \qquad \text{(S2-1)}$$

$$\frac{\partial Actin_m}{\partial t} = A_{on} Bni1_m Bud6_m Actin_c - A_{off} \frac{K_m}{K_m + Spa2_m} Actin_m \qquad \text{(S2-2)}$$

$$\frac{\partial Spa2_m}{\partial t} = \nabla^2 Spa2_m + S_{on} Spa2_c Actin_m - S_{off} Spa2_m \qquad \text{(S2-3)}$$

$$\frac{\partial Bud6_m}{\partial t} = \nabla^2 Bud6_m + B6_{fb} Spa2_m Bud6_c + B6_{on} Cdc42_m Bud6_c - B6_{off} Bud6_m \qquad \text{(S2-4)}$$

S2 Table lists the biochemical species in the system. S3 Table lists the stochastic formulated reactions, rates and references for these interactions. S4 Table lists the parameters and their default values for the model. S9 Fig and S10 Fig show visualizations of Bud6 and Spa2 on the membrane from a sample simulation of the model. S11 Fig shows the results from a parameter sweep (B_{fb}/B_{on} and K_m) of the original model to complement the parameter sweep described in (1). The results show a similar parameter map as before, in which the area of both successful tracking and tight polarization (purple region in S11A Fig) is a narrow band in the parameter space. S12 Fig shows a parameter sweep using the Bud6-related parameters $B6_{fb}$ and $B6_{on}$. This figure shows that the two criteria of tracking and tight polarization are only satisfied if the ratio of $B6_{on}$ to $B6_{fb}$ is in a tight range (diagonal of S12A Fig). It also demonstrates that if we constrain the amount of Bud6 on the membrane to be 50% of the total in the system, we obtain a unique region of parameter space.

Model Cell Geometry.

The model was implemented on a 1-D periodic domain (circular membrane) of diameter 8 microns. This domain was discretized into 160 equally sized voxels of circumferential length π/20 microns. We assumed that protein in the cytoplasm diffuses much (>1000-fold) faster than on the membrane, and thus we approximated the cytoplasm as well-mixed.

Supplemental References

1. Lawson MJ, Drawert B, Khammash M, Petzold L, Yi T-M. Spatial stochastic dynamics enable robust cell polarization. PLoS Computational Biology. 2013;9:e1003139.

2. Altschuler SJ, Angenent SB, Wang Y, Wu LF. On the spontaneous emergence of cell polarity. Nature. 2008;454(7206):886-9.
