# Supplementary material for: A positive feedback loop involving the Spa2 SHD domain contributes to focal polarization
Source: PLoS One. 2022 Feb 8;17(2):e0263347. doi: 10.1371/journal.pone.0263347 (PMC8824340; doi:10.1371/journal.pone.0263347)
Supplement: S1 Raw images — (PDF) [file pone.0263347.s023.pdf]

# S1 Raw Images File

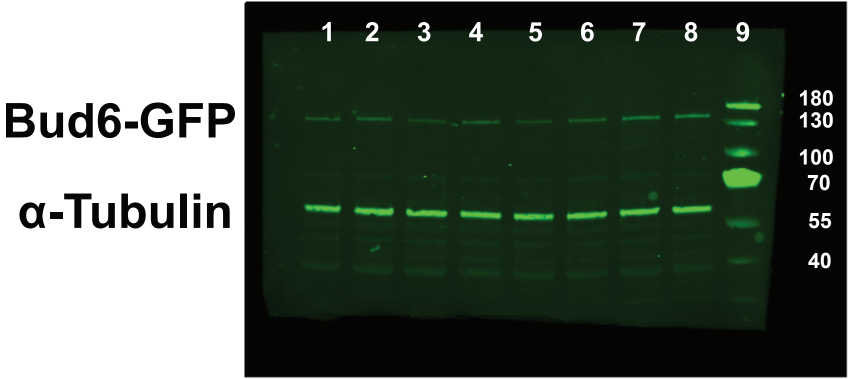


## S5 Fig original image.

Lanes 1 to 9 are protein extracts from the following strains (as described in S5 Fig): WT, *655Δ*, *NΔ200*, *SDR1*$^{4A}$, *SDR12*$^{4A}$, *msb3Δ msb4Δ*, *sec4-8*, and MW markers (size of markers are shown on side). Western blots were performed with anti-GFP and anti-α-tubulin antibodies, and band fluorescence was imaged using the LI-COR Odyssey system.
